# Research progress on the role and mechanism of circadian clock gene *PER1* in the occurrence and development of multiple diseases

**DOI:** 10.3389/fimmu.2025.1715457

**Published:** 2025-12-10

**Authors:** Yuxiang Zhao, Yujin Li, Tingting Lu, Xinyue Huang, Kerui Chen, Lina Tan, Lihua Gao

**Affiliations:** 1 Departments of Dermatology, The Third Xiangya Hospital, Central South University, Changsha, Hunan, China; 2 Medical Ozone Research Center of Central South University, Changsha, Hunan, China

**Keywords:** period 1, circadian rhythm, immune system diseases, tumors, cardiovascular diseases, nervous system diseases, metabolic disorders

## Abstract

Circadian rhythm, an inherent 24-hour periodic rhythm in organisms, is regulated by circadian clock genes. As a key component of the transcription-translation feedback loop, the core circadian gene period (*PER) 1*, not only maintains circadian rhythm homeostasis but also plays a significant role in the pathophysiological processes of various human diseases. This review summarizes the biological characteristics and regulatory mechanisms of *PER1*, as well as its roles and molecular mechanisms in cardiovascular diseases, nervous system diseases, metabolic disorders, immune-related diseases, and tumors. In cardiovascular diseases, *PER1* helps regulate blood pressure, renal function, and vascular inflammation. In the nervous system, it influences ischemic brain injury, sleep homeostasis, and neurodegenerative diseases. In metabolic disorders, *PER1* modulates endocrine function, glucose-lipid metabolism, and energy balance. In immune-related diseases, it regulates immune cell functions and inflammatory signaling. In tumors, *PER*1 has tumor-suppressive effects, with low expression correlating to poor prognosis. This review highlights the critical role of *PER1* as a core circadian gene in maintaining physiological homeostasis and regulating disease progression, providing a comprehensive perspective for understanding its complex functions in physiological and pathological processes, offering new insights for developing precision therapies targeting *PER1* and its associated signaling pathways.

## 1 Introduction

Biological rhythms are widely present in various life activities in nature. They are endogenous rhythmic life activities that organisms have gradually formed during their occurrence and evolution over hundreds of millions of years to adapt to environmental changes, which are similar to the periodic changes of the natural environment. In short, biological rhythms are the core mechanism for organisms to adapt to the environment, achieving dynamic balance between physiology, behavior and the environment through precise time regulation. Biological rhythms include annual rhythms, monthly rhythms and circadian rhythms. Circadian rhythm is an important biological rhythm, which is the inherent 24-hour periodic rhythm in organisms and is maintained by a highly conserved molecular rhythm device - the biological clock (1). The biological clock is an intrinsic time-regulating system that influences the physiology and behavior of organisms by regulating periodic changes in gene expression (2, 3). A variety of physiological functions of organisms, such as sleep-wake cycles, metabolism, endocrine, immunity, body temperature and cognition, as well as the functional operation of various systems, are all precisely regulated by circadian rhythms (4–6).

The central regulatory hub of circadian rhythms resides within the suprachiasmatic nucleus (SCN) of the hypothalamus. Functioning as the “master clock,” the SCN receives photic inputs via the retinohypothalamic tract, thereby achieving synchronization with the Earth’s 24-hour light-dark cycle. Through the secretion of neurotransmitters (e.g., vasoactive intestinal peptide), the SCN orchestrates rhythmicity across peripheral tissues throughout the organism (7). Beyond the SCN, autonomous molecular circadian oscillators are present in most peripheral tissues (such as the liver and lungs), which maintain synchronization with the central clock through neurohumoral signaling (8). At the molecular level, circadian rhythms are driven by a transcription-translation feedback loop (TTFL) consisting of core clock genes, including brain and muscle arnt-like 1 (*BMAL1)*, circadian locomotor output cycles kaput *(CLOCK), PER, and* cryptochrome (*CRY)*. These genes generate molecular oscillations with a periodicity of approximately 24 hours via time-delayed feedback regulation (9).

**Figure 1:**
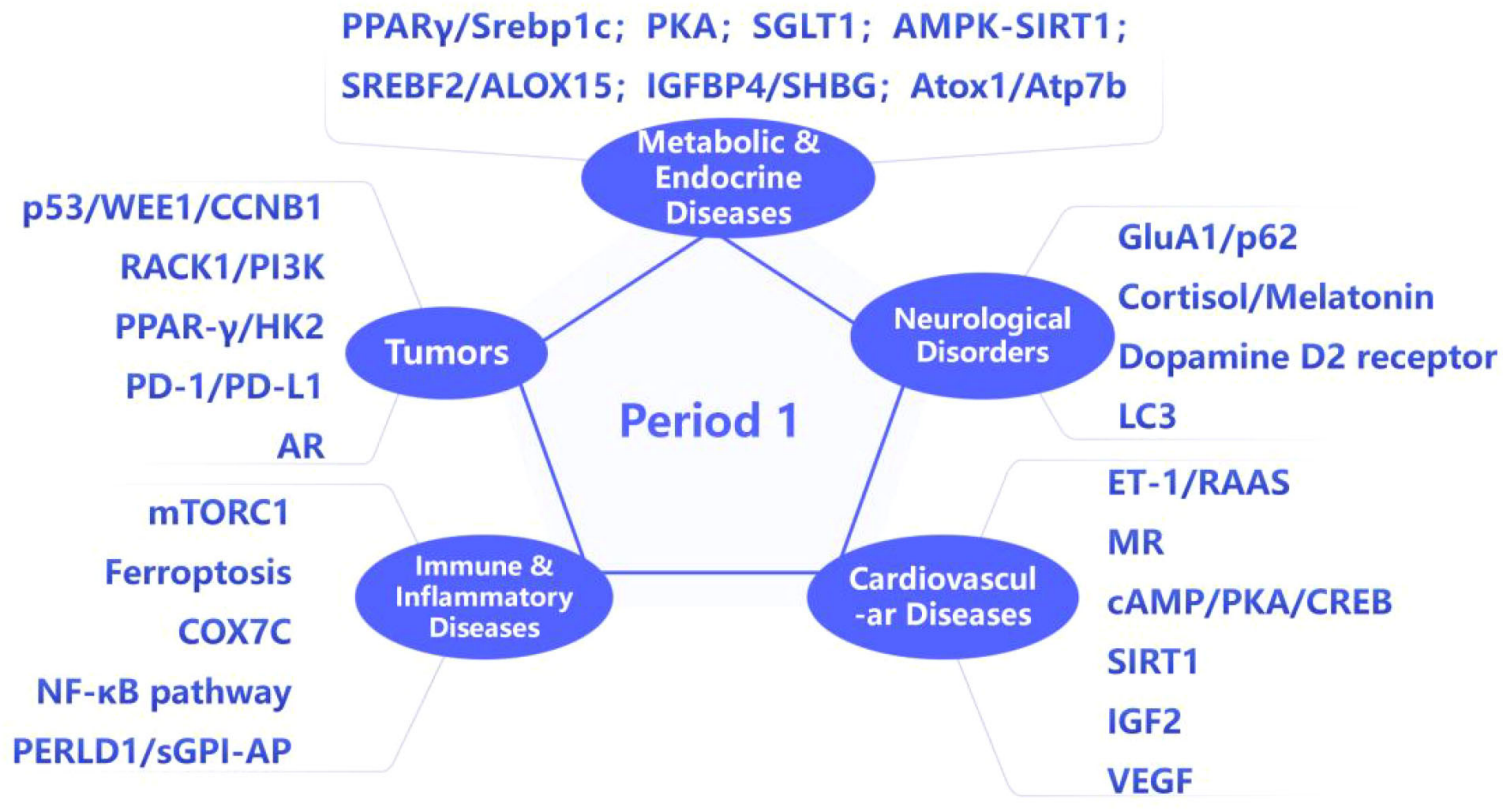
Role and mechanism of *PER1* in diseases.

The discovery of the *PER1* gene, a core circadian clock regulatory gene, can be traced back to homologous studies on the Period gene in Drosophila, making it one of the earliest identified core clock genes (10). As a crucial component of the circadian clock, *PER1* not only participates in maintaining normal circadian rhythms but also plays a significant role in the pathogenesis of various diseases (11). In recent years, the roles of *PER1* in cancer, cardiovascular diseases, metabolic disorders, neurodegenerative diseases, and immune-related diseases have been gradually unveiled. Its molecular mechanisms involve multiple levels, including cell cycle regulation, metabolic pathway intervention, and immune response modulation, thus emerging as a key target in interdisciplinary research (**Figure 1**). Starting from the biological functions of *PER1*, this article reviews its roles, pathophysiological significance, and related molecular mechanisms in cardiovascular diseases, neurodegenerative diseases, metabolic disorders, immune-related diseases, and cancer. Additionally, we distinguished levels of evidence and cross-species conservation of mechanism, aiming to provide new insights and targets for disease prevention and treatment.

## 2 Biological characteristics and regulatory mechanisms of PER1

### 2.1 Core components of the circadian negative feedback loop

*PER1* is localized on human chromosome 17p13.1 and encodes a nuclear protein containing a PAS domain. As a key component of the TTFL, *PER1* works in conjunction with other circadian clock genes to maintain the body’s circadian rhythm. The core of the molecular mechanism underlying the circadian clock is an autonomous oscillatory system composed of TTFL, in which PER proteins, together with CLOCK, BMAL1, and CRY proteins, form a core regulatory network (12, 13). Period circadian regulator serves as a core regulatory factor in the negative feedback loop of the mammalian circadian clock, and together with CLOCK, BMAL1, CRY RAR-related orphan receptors (RORs), and nuclear receptor subfamily 1 group D member 1 (NR1D1), it regulates the circadian rhythmicity of physiological activities in the organism.

As a pair of transcriptional activators, CLOCK and BMAL1 form a CLOCK-BMAL1 heterodimer, which binds to the E-box in the promoter region to activate the transcription of *PER* and *CRY* genes. When PER and CRY proteins reach a certain concentration in the cytoplasm, they form a dimer, translocate into the nucleus, and then inhibit the transcriptional activity of the CLOCK-BMAL1 heterodimer. This negative feedback mechanism ensures the timely suppression of *PER* and *CRY* transcription, thereby maintaining the stable operation of the circadian clock (14–16). At the level of species evolution, the *PER* gene family has generated multiple paralogous genes (e.g., *PER1/PER2/PER3*) in vertebrates through gene duplication events. Among them, *PER1* plays a critical role in maintaining rhythm plasticity, and its cis-regulatory elements exhibit characteristics of rapid evolution across different lineages (17, 18).

### 2.2 Rhythmicity and tissue-specificity of *PER1* expression

The expression of *PER1* exhibits variations across individuals of different ages and genders, with distinct temporal fluctuations throughout a 24-hour period (19). Accumulating evidence indicates that *PER1* expression follows a well-defined circadian pattern. For example, in human peripheral blood, *PER1* expression displays a diurnal phase, peaking at 9:00 AM and remaining low during nighttime (11), Similarly, *PER1* messenger RNA (mRNA) levels are downregulated between Zeitgeber time (ZT) 0–2 and reach maximal expression between ZT 12–14 (20).

The expression of *PER1* is not confined to SCN, the primary circadian pacemaker, but is widely distributed across peripheral tissues and cells. Its expression levels and functional roles are tissue-specific, contributing to the specific regulation of local tissues. For instance, within the SCN, *PER1* can respond to light stimuli and participate in the regulation of circadian rhythms. In peripheral tissues such as the liver and kidneys, *PER1* controls physiological rhythms by regulating gene expression, thereby influencing metabolic processes, body temperature, blood pressure, and other physiological functions. In tumors, the low expression of *PER1* may be associated with the regulation of tumor cell proliferation, invasion, and apoptosis (21–23).

### 2.3 Regulatory mechanisms of *PER1*

The stability of PER1 is regulated by casein kinase 1 (CK1), which modulates its degradation rate through phosphorylation of specific domains (e.g., D1 and D2) in PER1, thereby determining the length of the circadian period. The phosphorylation status of PER1 (such as CK1-mediated phosphorylation) influences its stability and the transcription of downstream genes, which holds significant pathological implications in various diseases (24, 25). Far upstream element-binding protein 1 maintains circadian rhythm stability by regulating the rhythmic expression of PER1 protein; its depletion leads to disruptions in the oscillatory pattern of PER1 protein (26). The mRNA expression of *PER1* is negatively regulated by fragile X mental retardation protein (FMRP), and the absence of FMRP results in disturbances in the oscillation of PER1 protein (27).

Studies have demonstrated that the expression of *PER1* is significantly influenced by external environmental factors (such as light exposure and nutritional status) and genetic variations. For example, light can regulate *PER1* expression in the SCN via the retinohypothalamic tract, while time-restricted feeding affects the oscillation of *PER1* in peripheral tissues by modulating intestinal hormones and metabolic signals (28–31). *PER1* is also dynamically regulated by hormones, receptors, and other metabolites. Specifically, glucocorticoid receptor (GR) and mineralocorticoid receptor (MR) bind to the *PER1* promoter in a periodic manner, dynamically regulating *PER1* transcription through the formation of homodimers or heterodimers (32); acute stimulation with glucocorticoids can directly induce *PER1* mRNA expression (33–35); and cellular iron levels can also modulate *PER1* expression (36).

In summary, PER1 protein exhibits a distinct circadian expression pattern, with its expression levels precisely regulated by phosphorylation, epigenetic modifications, external environment, hormones, and receptor signals. It exerts tissue-specific functions in both central and peripheral tissues, participating in the regulation of fundamental life processes such as cellular metabolism, proliferation, and differentiation, and thus holds significant implications in various diseases.

## 3 Role and mechanism of *PER1* in diseases

Recent studies have revealed that abnormal expression or functional impairment of *PER1* is closely associated with the occurrence and progression of various diseases. In cardiovascular diseases, *PER1* regulates blood pressure and renal function, and is involved in the anti-inflammatory mechanism of vascular injury. In the nervous system, *PER1* regulates ischemic brain injury, sleep homeostasis, and associates with neurodegenerative diseases. In metabolic disorders, *PER1* modulates endocrine function, glucose and lipid metabolism, as well as energy balance. In immune-related diseases, it regulates immune cell functions and inflammatory signaling pathways. In tumors, *PER1* exerts tumor-suppressive effects and its low expression links to poor prognosis.

### 3.1 Cardiovascular diseases

*PER1*, a core component of the circadian clock system, is increasingly recognized as a key regulator of cardiovascular physiology and pathology. Its rhythmic expression in cardiovascular tissues (e.g., heart, blood vessels, and regulatory organs like the kidneys) integrates circadian rhythms with cardiovascular function, and its dysregulation contributes to the development and progression of various cardiovascular diseases (**Table 1**).

**Table 1:** Regulatory directions and mechanisms of action of *PER1* in cardiovascular diseases.

Type of regulation	Related signaling molecules/pathways	Specific mechanisms	Evidence level	Cross-species conservation of mechanism	References
Blood Pressure, Sodium Metabolism, and Renal Function	ET-1	Regulation of renal sodium handling and blood pressure rhythm through modulating the expression and activity of ET-1 may be involved in the regulation of hypertension progression via the RAAS	Preclinical evidence (animal model)	Partially conservative	(37, 38)
	MR	PER1 and MR may have a close regulatory relationship	Preclinical evidence (animal and cell models)	Conservative	(24, 25, 39, 41)
	cAMP/PKA/CREB	GPR183 disrupts circadian rhythm signaling by inhibiting *PER1* expression and promotes endothelial senescence and dysfunction through the cAMP/PKA/CREB pathway	Preclinical evidence (animal model)	Partially conservative	(42)
	Orexin system genes	*PER1* and orexin system genes exhibit coordinated regulation of their expression, which may influence the pathogenesis of sleep apnea syndrome	Preclinical evidence (animal model)	Conservative pending verification	(43)
Anti-Inflammatory Mechanism of Vascular Injury	SIRT1 and other rhythmic genes	*PER1* may be involved in activating SIRT1 and other rhythm-related genes, thereby enhancing antioxidant and anti-inflammatory capacities	Preclinical evidence (animal model)	Conservative pending verification	(45)
	IGF2 anti-inflammatory pathway	*PER1* indirectly regulates the IGF2 anti-inflammatory pathway by maintaining normal SCN function or synergizing with other rhythmic genes	Preclinical evidence (animal model)	Conservative pending verification	(46)
	VEGF pathway	The overexpression of *PER1* inhibits macrophage M1 polarization and the release of pro-inflammatory factors, as well as by activating the VEGF pathway	Preclinical evidence (animal and cell model)	Partially conservative	(47)

#### 3.1.1 Regulation of blood pressure, sodium metabolism, and renal function

The *PER1* gene plays a critical role in regulating blood pressure, sodium metabolism, and renal function, particularly in salt-sensitive hypertension and renal injury. Studies have shown that Dahl salt-sensitive rats with *PER1* gene knockout (KO) exhibit elevated blood pressure, increased renal expression of endothelin-1 (ET-1), and exacerbated renal damage (37, 38). In *PER1^−^/^−^* rats, plasma aldosterone levels and MR expression are elevated; specifically, KO of *PER1* in distal nephrons and collecting ducts leads to increased aldosterone levels and enhanced renal Na^+^ retention (39). *PER1* gene knockout displays significant sex-dependent differences across various animal models (e.g., mice and rats): male mice show a more pronounced response to KO of *PER1*, characterized by more severe hypertension and renal injury, whereas female mice remain unaffected (40).Mechanistically, *PER1* influences renal sodium handling and blood pressure rhythms by regulating the expression and activity of ET-1, thereby antagonizing salt load-induced vascular contraction and fibrosis. This suggests that *PER1* may be involved in the progression of hypertension by regulating the renin-angiotensin-aldosterone system (RAAS). Studies have shown that the interaction between PER1 and CK1 affects its phosphorylation and degradation, and CK1 regulates MR function by directly phosphorylating MR and indirectly phosphorylating MR co-regulators, implying a potential close regulatory relationship between PER1 and MR (24, 25, 41). Furthermore, studies have found that GPR183 disrupts circadian rhythm signaling by inhibiting *PER1* expression, thereby promoting endothelial senescence and dysfunction through the cyclic 3’,5’-adenosine monophosphate (cAMP)/protein kinase A (PKA)/cAMP-response element binding protein (CREB) signaling pathway (42). In addition, in hypertension models, *PER1* and the orexin system genes exhibit coordinated regulation of expression, which may influence the pathogenesis of sleep apnea syndrome (43).

#### 3.1.2 Involvement in the anti-inflammatory mechanism of vascular injury

*PER1* is involved in the anti-inflammatory mechanism of vascular ischemic injury. Liposomal prednisolone can induce the translocation of glucocorticoid (GC) receptors to the nucleus and upregulate the expression of *PER1* mRNA, indicating that *PER1* may participate in the anti-inflammatory mechanism of liposomal prednisolone in renal ischemia-reperfusion injury in rats (44). *PER1* enhances myocardial tolerance to ischemia by maintaining circadian rhythms. In myocardial ischemia-reperfusion injury, KO of *PER1* and *PER*2 genes impairs antioxidant and anti-inflammatory capacities, thereby exacerbating myocardial damage. The underlying mechanism is that *PER1*/*PER*2 double-knockout mice, due to circadian rhythm disruption, fail to activate rhythmic genes such as sirtuin 1 (*SIRT1)*, leading to reduced antioxidant and anti-inflammatory capabilities (45). *PER1* also contributes to the SCN-mediated cardioprotective mechanism. Studies have demonstrated that disruption of SCN function can induce the polarization of macrophages toward an anti-inflammatory phenotype by upregulating insulin-like growth factor 2 (IGF2), thereby improving cardiac repair after myocardial infarction. This mechanism may involve *PER1* indirectly regulating the IGF2-mediated anti-inflammatory pathway through maintaining normal SCN function or synergizing with other rhythmic genes (46). In a mouse model of hindlimb ischemia (HLI), reduced expression of *PER1* in skeletal muscle is associated with abnormal macrophage polarization (increased CD68) and decreased angiogenesis (reduced CD31). Overexpression of *PER1* alleviates myocyte injury and promotes blood flow recovery via mechanisms involving the inhibition of macrophage M1 polarization and proinflammatory factor release, while activating the vascular endothelial growth factor (VEGF) pathway. This finding provides a novel therapeutic target for peripheral arterial disease (47).

### 3.2 Neurological disorders

*PER1* exerts a pivotal role in neurological disorders, including ischemic brain injury, sleep homeostasis, and degenerative neuropathies, through multiple regulatory pathways such as circadian rhythm modulation, cell autophagy, oxidative stress, cellular apoptosis, and neurotransmitter systems (**Table 2**).

**Table 2:** Regulatory pathways/molecules, specific mechanisms of *PER1* in neurological diseases.

Types of neurological diseases	Related signaling molecules/pathways	Specific mechanisms	Evidence level	Cross-species conservation of mechanism	References
Ischemic Brain Injury	Genetic variants of *PER1* (rs2253820)	the rs225382 variant exacerbate the circadian rhythm of blood pressure	Preclinical evidence (animal model)	Conservative pending verification	(48)
	GluA1, p62	Deletion of *PER1* disrupts p62-mediated selective autophagic degradation of GluA1 and reduces autophagic activity	Preclinical evidence (animal model)	Conservative pending verification	(49)
	Autophagy-related molecules (such as LC3, etc.)	*PER1* deficiency disrupts the normal activity of the autophagic machinery (the basal LC3 levels were dramatically reduced with no changes in autophagic markers such as the LC3-II/LC3-I ratio)	Preclinical evidence (animal and cell models)	Partially conservative	(50, 51)
Sleep Homeostasis	*CRY1*	Coordinated changes in the co-expression of *PER1* and *CRY1* play a critical role in the disruption of sleep homeostasis after stroke	Clinical evidence (cohort study)	Conservative	(52)
	Cortisol, melatonin	In rats with ischemic stroke, *PER1* is associated with sleep-wake cycle disturbances, increased cortisol levels, and decreased melatonin secretion	Preclinical evidence (animal model)	Conservative	(53, 54)
PD	*PER1* gene variants (e.g., loss-of-function, rs2225380 variants)	Loss-of-function variants of *PER1* are associated with motor dysfunctions in PD, such as dyskinesia	Clinical evidence (cohort study)	Conservative pending verification	(58)
		The rs2225380 variant is positively correlated with PIGD subtype of sporadic PD	Clinical evidence (case-control study)		(59)
	Dopamine D2 receptor	Upregulation of *PER1* in the nucleus accumbens is associated with the activation of dopamine D2 receptors	Preclinical evidence (animal model)	Conservative	(60, 61)
AD	*PER1* gene polymorphism (rs3027178)	The G allele of rs3027178 confers a protective effect against AD	Clinical evidence (case-control study)	Conservative pending verification	(63)
	Autophagy-related molecules	KO of *PER1* accelerates hippocampal aging in aged mice, which is related to impaired autophagy and excessive accumulation of misfolded proteins	Preclinical evidence (animal model)	Conservative pending verification	(65)
Other neural functions	CREB	*PER1* influences time-dependent memory formation by regulating the daytime phosphorylation of CREB in the hippocampus	Preclinical evidence (animal model)	Partially conservative	(66, 67)
	SYT14	SYT14 deficiency leads to a significant upregulation of *PER1* in the hippocampus, accompanied by behavioral abnormalities	Preclinical evidence (animal model)	Conservative pending verification	(68)
	Ion transport and apoptosis signal-related molecules	It may exacerbate neural damage by affecting ion transport and apoptotic signaling, and is associated with abnormal synaptic vesicles	Preclinical evidence (animal model)	Conservative pending verification	(69)

#### 3.2.1 Maintaining autophagic activity in ischemic brain injury

In ischemic stroke, the enlargement of cerebral infarct volume has been associated with circadian rhythm disruption. Genetic variation at the rs2253820 locus of the *PER1* gene has been shown to exacerbate the circadian rhythm of blood pressure (48). In a mouse model of cerebral ischemia, KO of *PER1* eliminated the circadian variation in infarct volume, abolishing the protective effect observed during the active phase (nighttime). This was accompanied by impaired degradation of the glutamate ionotropic receptor AMPA type subunit 1 (GluA1) and reduced autophagic activity. In wild-type mice, ischemia during the active phase triggered sequestosome 1 (p62) - mediated selective autophagic degradation of GluA1 receptors, a process that was disrupted in the absence of *PER1*. These findings suggest that *PER1* mitigates excitotoxicity by maintaining circadian regulation of autophagy-dependent GluA1 degradation (49). A study showed that levels of the proapoptotic factors cytochrome c (Cyt c) and apoptotic protease activating factor 1 (Apaf1) were higher in *PER1*^−^/^−^ mice and basal LC3 levels were dramatically reduced. It is indicated that *PER1* deficiency may slow down the autophagic machinery, increasing neuronal susceptibility to cerebral ischemia (50). Consistently with this, another study reported that hippocampal neurons from *PER1*^−^/^−^ mice exhibited resistance to rapamycin-induced autophagy activation, as indicated by unchanged autophagic markers (e.g., LC3-II/LC3-I ratio), further underscoring the indispensable role of *PER1* in maintaining basal autophagic activity (51).

#### 3.2.2 Regulation of sleep homeostasis

Whereas inverse associations were observed with sleep latency, as well as α, β, and δ wave activities in the O1-A2 electrode derivation (52, 53). Moreover, coordinated changes in the co-expression of *PER1* and *CRY1* suggest that the core clock gene network plays a critical role in post-stroke sleep homeostasis disruption. In a rat model of ischemic stroke, pineal *PER1* mRNA and protein levels were significantly elevated, particularly in aged rats, which correlated with sleep-wake cycle disturbances, increased cortisol levels, and decreased melatonin secretion (54). In clinical populations, reduced *PER1* expression has been reported in individuals with chronic insomnia or those working night shifts, potentially linking circadian dysregulation to accelerated neurodegenerative processes, although the underlying mechanisms remain to be fully elucidated (55).

#### 3.2.3 Association of *PER1* with neurodegenerative diseases

Genetic variants of *PER1* have been directly linked to Parkinson’s disease (PD), particularly in the pathogenesis of motor dysfunctions (56, 57). Genetic analyses revealed significant enrichment of loss-of-function variants (e.g., missense mutations) in the *PER1* gene among PD cohorts, which were associated with dyskinesia. A whole-exome sequencing study confirmed that PD patients carrying deleterious *PER1* variants exhibited a higher incidence of dyskinesia, highlighting these variants as critical risk factors for PD-related motor symptoms (58). Gu et al. reported a significant association between clock genes and sporadic PD in the Chinese population, with the *PER1* variant rs2253820 showing a stronger positive correlation in postural instability and gait disorder (PIGD) subtypes (59). These findings underscore *PER1* as a susceptibility gene for PD.

Mechanistically, the variants of *PER1* may disrupt dopamine signaling or circadian output pathways, leading to neuronal dysfunction and progressive degeneration. This is supported by animal studies demonstrating that *PER1* upregulation in the nucleus accumbens is associated with dopamine D2 receptor activation, potentially modulating neurotransmitter release and influencing alcohol addiction behaviors (60, 61). Furthermore, *PER1* genotypes (e.g., AG carriers of the risk allele G) have been associated with depressive states and altered white matter integrity, which may manifest as comorbid symptoms in neurodegenerative diseases such as Alzheimer’s disease (AD). Brain imaging data showed that AG carriers had a higher prevalence of depression and microstructural damage in white matter regions such as the corpus callosum compared to AA homozygotes (62). These results suggest that *PER1* may contribute to the symptomatology of neurodegenerative diseases by regulating emotion-related signaling pathways (e.g., dopamine or glutamate systems).

*PER1* influences AD risk through genetic polymorphisms and plays a critical role in hippocampal aging and AD pathology by regulating glial function, amyloid metabolism, and autophagic pathways. A significant association was identified between the rs3027178 polymorphism in the *PER1* circadian gene and AD risk, with the G allele conferring a protective effect (63). Additionally, KO of *PER1* accelerates age-related hippocampal changes in 24-month-old mice, including microglial morphological alterations, Aβ and lipofuscin deposition, and presenilin overexpression. These alterations are attributed to impaired autophagy and excessive accumulation of misfolded proteins in the hippocampus, thereby increasing neuronal vulnerability (64, 65).

In degenerative diseases, changes in *PER1* expression are not only associated with specific disorders but also broadly involved in regulating behavior, memory, and neural functions. *PER1* modulates time-dependent memory formation by gating the phosphorylation of CREB in the hippocampus (activated exclusively during the daytime), thereby linking circadian rhythms to learning efficiency (66, 67). Studies have shown that synaptotagmin 14 (*SYT14*) gene deficiency leads to a significant upregulation of *PER1* expression in the hippocampus, accompanied by behavioral alterations (e.g., hyperactivity), which are related to dysregulated neural signaling pathways. *PER1* may exacerbate neural damage by affecting ion transport and apoptotic signaling; ultrastructural studies further confirmed abnormal vesicle counts, suggesting an impact of *PER1* on synaptic function (68). This mechanism is particularly crucial in degenerative diseases, as neurodegenerative disorders often involve protein homeostasis imbalance and mitochondrial dysfunction (69).

### 3.3 Endocrine and metabolic diseases

*PER1* functions as a critical integrator of circadian rhythms and metabolic/endocrine signals, regulating glucose and lipid homeostasis, maintenance of energy balance, reproductive endocrine balance, and trace element metabolism (**Table 3**).

**Table 3:** Regulatory roles and specific mechanisms of *PER1* in metabolism, endocrinology, and copper metabolism.

Type of regulation	Role of *PER1*	Specific mechanisms	Evidence level	Cross-species conservation of mechanism	References
Lipid Metabolism	Upregulate the expression of target genes	Overexpression can upregulate the expression of PPARγ and its target genes, and promote the expression of adipogenic genes (including Srebp1c)	Preclinical evidence (animal model)	Conservative	(70, 71)
	Influence hepatic lipid accumulation	KO can reduce hepatic lipid accumulation and protect mice from ethanol-induced liver injury	Preclinical evidence (animal model)	Conservative pending verification	(70)
	Interact with hepatic enzymes and regulate bile acid synthesis	It directly interacts with key hepatic enzymes involved in bile acid synthesis and participates in the rhythmic biosynthesis of bile acids via the PER1/PKA-mediated phosphorylation pathway	Preclinical evidence (animal model)	Conservative pending verification	(72)
	Respond to stress stimuli	Fasting and high-fat stress can enhance *PER1* expression, thereby increasing fat absorption and accumulation	Preclinical evidence (animal model)	Conservative pending verification	(72)
	Association with serum indices	Serum triglyceride concentration is negatively correlated with *PER1* mRNA levels (and positively correlated with *CLOCK* mRNA levels)	Preclinical evidence (animal model)	Conservative pending verification	(73)
Glucose Metabolism	Affect glucose absorption	It regulates the transcription of SGLT1 by modulating the activity of E-box elements in the SGLT1 promoter	Preclinical evidence (animal and cell model)	Partially conservative	(74)
	Correlation with insulin resistance	Downregulation of *PER1* may induce and exacerbates insulin resistance	Preclinical evidence (animal model)	Partially conservative	(75, 76)
	Impact hepatic circadian clocks	The rhythmic expression of *PER1* in the liver and pancreas affects glucose homeostasis	Preclinical evidence (animal model)	Partially conservative	(77, 78)
	Mutual regulation with blood glucose	Hyperglycemia can disrupt the rhythmic expression of *PER1* in the liver and olfactory bulb, while glucose restriction induces the expression of the circadian clock gene *PER1* via the AMPK-SIRT1 pathway	Preclinical evidence (animal model)	Conservative pending verification	(79, 80)
Energy Balance	Regulate feeding behavior and energy expenditure	Phosphorylation of PER1 may determine feeding rhythms	Preclinical evidence (animal model)	Conservative pending verification	(81)
		The selective induction of hepatic *PER1* during fasting is a crucial mechanism by which hepatocytes integrate internal circadian rhythms and external nutritional signals	Preclinical evidence (animal model)	Conservative pending verification	(28, 82, 83)
Endocrine and Copper Metabolism	Regulate PCOS	*PER1* mediates ferroptosis and lipid metabolism by inhibiting the SREBF2/ALOX15 signaling pathway	Preclinical evidence (animal model)	Conservative pending verification	(84),
		Decreased expression of *PER1* and *PER2* can induce hyperandrogenism in rats	Clinical evidence (case-control study) and preclinical evidence (animal and cell models)	Conservative	(85)
	Modulate copper metabolism	Overexpression can alleviate nephrotoxicity by upregulating Atox1	Preclinical evidence (animal model)	Conservative pending verification	(86)
		Overexpression may exacerbate Cu-induced hepatotoxicity by downregulating Atp7b	Preclinical evidence (animal and cell models)	Conservative pending verification	(87)

#### 3.3.1 Regulation of lipid metabolism

The *PER1* gene can influence fat accumulation by regulating adipocyte differentiation and metabolism. Overexpression of *PER1* upregulates the expression of peroxisome proliferator-activated receptor - gamma (PPAR-γ) and its target genes, while *PER1* deletion protects mice from ethanol-induced liver injury by reducing hepatic lipid accumulation (70). *PER1* promotes the expression of adipogenic genes (including sterol regulatory element-binding protein 1c), thereby enhancing lipid synthesis (71). Similarly, studies by GE et al. revealed that *PER1* directly interacts with key hepatic enzymes involved in bile acid synthesis, such as cholesterol 7α-hydroxylase and sterol 12α-hydroxylase. The rhythmic biosynthesis of bile acids is associated with the activity and instability of bile acid synthases via the PER1/PKA-mediated phosphorylation pathway. Both fasting and high-fat stress can enhance *PER1* expression, thereby increasing fat absorption and accumulation (72). Research has also found that serum triglyceride concentrations are positively correlated with *CLOCK* mRNA levels but negatively correlated with *CRY2* and *PER1* mRNA levels (73). These studies indicate that *PER1* acts as an energy regulator, controlling daily fat absorption and accumulation.

#### 3.3.2 Regulation of glucose metabolism

*PER1* can affect glucose homeostasis by regulating insulin secretion and glucose uptake. It modulates the transcription of the sodium-glucose cotransporter 1 (SGLT1)—a glucose transporter—by regulating the activity of E-box elements in the SGLT1 promoter, thereby influencing glucose absorption (74). YAMAOKA et al. demonstrated that cold exposure in adipose tissue inhibits *PER1* expression, and downregulation of *PER1* may induce insulin resistance by impairing insulin signaling pathways (75). Xu et al. found that deletion of *PER1* and *PER2* exacerbates diet-induced insulin resistance and glucose intolerance, accompanied by aggravated hepatic inflammatory responses and metabolic dysregulation (76). FIGUEROA et al. reported that taurine improves obesity and diabetes by restoring the rhythmic expression of *PER1* in pancreatic β-cells, suggesting that *PER1* may serve as a potential target for taurine-based therapies in obesity and diabetes (77). Increased nuclear expression of *PER1* can disrupt hepatic circadian clocks, further exacerbating glucose homeostasis imbalance (78). While *PER1* influences blood glucose levels, blood glucose itself can regulate *PER1* expression in tissues: hyperglycemia can disrupt the rhythmic expression of *PER1* in the liver and olfactory bulb, leading to behavioral abnormalities (79), whereas glucose restriction induces the expression of the circadian clock gene *PER1* via the AMP-activated protein kinase (AMPK) - SIRT1 pathway (80).

#### 3.3.3 Maintenance of energy balance

The *PER1* gene is also involved in regulating the body’s energy balance. Through interactions with the circadian clock system, it influences multiple aspects such as basal metabolic rate, food intake, and energy expenditure. Studies have shown that phosphorylation of PER1 determines feeding rhythms in mice, and S714 in hPER1 is a key site driving the rhythm of food intake behavior, playing a critical role in the physiological optimization of feeding behavior and energy consumption (81). Additionally, research indicates that time-restricted feeding may improve metabolic function in obese adult mice by reducing the circadian clock genes *PER1* and *PER2* in the liver. The underlying mechanism may involve the selective induction of hepatic *PER1* during fasting; mice lacking hepatic *PER1* fail to initiate autophagic flux, ketogenesis, and lipid accumulation. This suggests that the induction of *PER1* may be an important mechanism by which hepatocytes integrate internal circadian rhythms and external nutritional signals to promote the appropriate utilization of calories (28, 82, 83).

#### 3.3.4 Regulation of endocrine and copper metabolism

A study suggested that *PER1* promotes ferroptosis and dysfunctional lipid metabolism in granulosa cells in polycystic ovary syndrome (PCOS) by inhibiting sterol regulatory element-binding factor 2 (SREBF2)/arachidonate 15-lipoxygenase (ALOX15) signaling pathway (84). However, another study revealed that decreased *PER1* and *PER2* promoted androgen excess via insulin-like growth factor-binding protein 4 (IGFBP4) and sex hormone binding globulin (SHBG) in the liver (85).

In addition, overexpression of *PER1* can reduce intracellular copper accumulation and alleviate nephrotoxicity by upregulating the copper chaperone protein antioxidant 1 copper chaperone (Atox1) (86). However, one study has found that the overexpression of *PER1* may exacerbate Cu-induced hepatotoxicity by downregulating Cu transporter Atp7b in Hepa1–6 cells (87). We hypothesize that *PER1* may exert distinct regulatory effects on copper metabolism across different tissues.

### 3.4 Immune and inflammatory-related diseases

*PER1* regulates immune cell functions, inflammatory factor expression, and signaling pathways, thereby playing a critical role in immune and inflammatory responses (**Table 4**), with significant implications particularly in the pathogenesis of inflammatory bowel disease and allergic airway inflammation.

**Table 4:** Regulatory mechanisms of *PER1* in immune cell function and inflammatory signaling pathways.

Regulatory category	Cell type	Related signaling molecules/pathways	Specific mechanisms	Evidence level	Cross-species conservation of mechanism	References
Regulation of the function of immune cells	Naive CD4^+^ T lymphocytes	mTORC1	Regulation of *PER1* expression inhibits mTORC1 and suppresses Th1 polarization through adrenergic and glucocorticoid stress signaling pathways	Preclinical evidence (cell model)	Conservative pending verification	(88)
	Splenic lymphocytes	Ferroptosis	KO of both *PER1* and *PER*2 genes induces ferroptosis, leading to a decrease in immune cell counts, architectural damage of lymphoid tissues, and compromised immune function	Preclinical evidence (animal model)	Conservative pending verification	(89)
	Treg Cell	COX7C	Decreased *PER1* expression and impaired immunosuppression during circadian disruption exacerbate autoimmune diseases (e.g. uveitis)	Clinical evidence (case-control study) and preclinical evidence (animal and cell models)	Conservative	(90)
	NK Cell	IFN-γ, Perforin,Granzyme B	Regulates NK cell rhythms and mediates circadian expression of IFN-γ, perforin and granzyme B	Preclinical evidence (animal model)	Conservative pending verification	(91)
	Macrophage	TNF-α,PPAR-γ,Ccr2,IL-1β,IL-6,CCL2	Inhibits M1 polarization and promotes M2 polarization; *PER1*/*PER2* mutations increase macrophage pro-inflammatory activation; interacts with PPAR-γ to reduce hepatic macrophage recruitment; inhibits inflammatory signaling pathways and enhances cellular reprogramming	Preclinical evidence (animal and cell models)	Partially conservative	(47, 76, 92, 93)
	B Cell	-	The expression dynamics of *PER1*^Venus^ reporter gene increased dramatically during the transitional period of B cell development	Preclinical evidence (animal model)	Conservative pending verification	(94)
Regulation of inflammatory signaling pathways	Serum and liver	PPAR-γ, Ccr2, TNF-α, IL-1β, IL-6, CCL2	*PER1* may enhance PPAR-γ-mediated transcriptional repression of Ccr2 and reduce inflammatory factor expression	Preclinical evidence (animal and cell models)	Partially conservative	(92)
	IL-1β-induced mandibular condylar chondrocytes	IL-1β, NF-κB, MMP13	Regulation of IL-1β-induced MMP13 expression through the NF-κB pathway	Preclinical evidence (animal and cell models)	Conservative pending verification	(95)
	Spinal cord astrocytes	p38, JNK1, NF-κB, CCL2, IL-6	Regulation of CCL2 and IL-6 production via activation of the p38, JNK1, and NF-κB signaling pathways	Preclinical evidence (cell model)	Conservative pending verification	(96)

#### 3.4.1 Regulation of immune cell functions

In T cells, the stress signaling pathways mediated by adrenergic and glucocorticoid hormones inhibit mTORC1 in naive CD4^+^ T cells by regulating the expression of the circadian rhythm gene *PER1*, thereby suppressing Th1 polarization (88). Double KO of *PER1* and *PER2* induces ferroptosis in splenic lymphocytes, leading to a reduction in the number of splenic immune cells, structural damage, and impairment of immune function (89). Circadian clock disturbances impair the stability and function of Treg cells through *PER1*-related cytochrome c oxidase subunit 7C (COX7C) - dependent mitochondrial metabolic processes, exacerbating diseases such as autoimmune uveitis (90), indicating that *PER1* is a key factor in maintaining the stability and function of regulatory T (Treg) cells. *PER1* plays an important regulatory role in the rhythm of natural killer (NK) cells and can mediate the circadian expression of interferon-gamma (IFN-γ), perforin, and granzyme B (91). Many studies have demonstrated a close association between PER1 and macrophages. Mutations in *PER1* and *PER2* disrupt circadian rhythms and boost macrophage inflammation (71). *PER1* interacts with PPAR-γ to curb hepatic macrophage recruitment (92), shifts macrophage polarization from M1 to M2 (41), and reins in macrophage inflammatory signaling to spur cell reprogramming (93). In addition to the above immune cells, B cells are also regulated by clock elements. Dynamic regulation of *PER1*^Venus^ levels has been observed during B cell development, with a sharp increase in reporter gene expression during the transitional phase (94).

#### 3.4.2 Regulation of inflammatory signaling pathways

*PER1* can also participate in autoimmune pathological processes by regulating inflammatory signaling pathways. Studies have found that inflammatory cytokines such as tumor necrosis factor - alpha (TNF-α), interleukin (IL) - 1β, IL-6, and chemokine (C-C motif) ligand 2 (CCL2) are increased in the serum and liver of *PER1*^-^/^-^ mice. The underlying mechanism may be that *PER1* interacts with PPAR-γ in the promoter region of the CC chemokine receptor 2 (Ccr2) gene, thereby enhancing PPAR-γ-mediated transcriptional inhibition of Ccr2 and attenuating excessive innate immune responses in endotoxin-induced liver injury (92). *PER1* can regulate IL-1β-induced matrix metalloproteinase (MMP) 13 expression in mandibular condylar chondrocytes through the nuclear factor-kappa B (NF-κB) pathway (95). And *PER1* can regulate the production of CCL2 and IL-6 through the activation of p38 mitogen-activated protein kinase (p38), c-Jun N-terminal kinase 1 (JNK1), and NF-κB in spinal astrocytes (96).

#### 3.4.3 Participate in the pathogenesis of inflammatory diseases

Circadian clock genes (including *PER1*) are downregulated in intestinal tissues and peripheral blood mononuclear cells of patients with inflammatory bowel disease (IBD) (97). *PER1* plays a critical role in maintaining intestinal barrier function. Studies have shown that intestinal mucosal barrier function is weakened in *PER1*/*PER*2 double-KO mice, accompanied by exacerbation of chronic colitis. Colon biopsy results reveal significantly upregulated wee1-like protein kinase (WEE1) mRNA levels and enhanced expression of cellular inhibitor of apoptosis protein 2 (cIAP2). The potential mechanism suggests that double-KO of *PER1*/*PER*2 triggers impaired cell division during proliferation via Wee1, leading to upregulation of anti-apoptotic pathways (98). In an ovalbumin-induced allergic airway inflammation model, *PER1* protein expression is significantly upregulated in mouse lung tissues. *PER1* may serve as a negative regulator of melatonin against Th2-type airway inflammation (99). Additionally, research has identified a functional PER1-like domain-containing protein 1 (PERLD1). PERLD1 haplotype can alter the sensitivity of peripheral blood mononuclear cells (PBMCs) through influencing soluble glycosylphosphatidylinositol anchor protein (sGPI-AP) levels, potentially contributing to individual susceptibility to allergic asthma (100).

### 3.5 Tumor

#### 3.5.1 Low expression of *PER1* and its prognostic relevance

*PER1* is significantly downregulated in various malignant tumor tissues, including breast cancer, lung cancer, prostate cancer, and oral squamous cell carcinoma (101–103). The downregulation of *PER1* is associated with poor prognosis: patients with high *PER1* expression in gastric cancer exhibit prolonged survival (P = 0.0028) (104). In breast cancer tissues, high *PER1* expression is correlated with longer overall survival and recurrence-free survival (HR: 0.78, 95% CI: 0.63–0.97) (105). In ovarian cancer, low *PER1* expression is linked to reduced overall survival, particularly in early-stage (I+II) patients where low expression indicates poor prognosis (106).

#### 3.5.2 Core mechanisms of *PER1* in tumor suppression

*PER1* plays an important role in various tumors, such as cholangiocarcinoma, glioma, oral squamous cell carcinoma, nasopharyngeal carcinoma, prostate cancer, and gastric cancer (**Table 5**). Its mechanisms involve cell proliferation, apoptosis, cell cycle progression, metabolism, immune regulation, etc.

**Table 5:** Role of *PER1* and related regulatory molecules/pathways in different types of tumors.

Type of tumor	Mechanism of action	Regulatory molecules/pathways	Evidence level	Cross-species conservation of mechanism	Reference
Cholangiocarcinoma	Overexpression inhibits cell proliferation via upregulating cell cycle regulators, with increased G2/M and S phase cells and reduced G1 phase cells	WEE1, CREB phosphatase 1, CRE-BP1	Preclinical evidence (animal and cell model)	Conservative	(114)
Glioma	*PER1* may be attributable to enhanced CHK2-p53 signaling and proapoptotic processes	CHK2-p53	Preclinical evidence (cell model)	Conservative pending verification	(111)
OSCC	KO promotes cell growth, proliferation, anti-apoptosis, migration and invasion; it inhibits glycolysis-mediated cell proliferation	Ki-67, MDM2, BCL-2, MMP2, MMP9; C-MYC, p53, BAX, TIMP-2; PER1/RACK1/PI3K, PI3K/AKT	Preclinical evidence (animal and cell models)	Conservative	(103, 116, 117, 119)
Nasopharyngeal Carcinoma	Overexpression reduces cell invasion and migration	-	Preclinical evidence (cell model)	Conservative pending verification	(117)
Prostate Cancer	It interacts with AR, serving as a negative regulator of AR activity to maintain hormonal homeostasis	AR	Preclinical evidence (animal and cell models)	Conservative	(101)
Breast Cancer	Downregulation of *PER1* in tumor cells increases tumor growth, but only at two specific times of the day	-	Preclinical evidence (animal and cell models)	Conservative	(110)
	Promoter methylation of the *PER1* correlates with c-erbB2 immunohistochemical reaction of > or = 2+ and has a strong inverse correlation with ER positivity		Clinical evidence	Partially conservative	(108)
Trastuzumab-resistant Gastric Cancer Cells	It forms a complex with PPAR-γ, promotes the upregulation of HK2, and enhances glycolytic activity	PPAR-γ, HK2	Preclinical evidence (animal and cell models)	Conservative	(120)
Ovarian Cancer	Expression levels are associated with immune infiltration and involved in immune evasion	Neutrophils, Treg cells, and M2-type macrophages (their infiltration is positively correlated with *PER1* expression)	Clinical evidence (cohort study)	Conservative pending verification	(106)
Endometrial Cancer	Overexpression promotes the expression of immune factors and the upregulation of immune checkpoints, and may inhibit tumor invasion by activating immune responses	TNF-α, IL-6, PD-1/PD-L1	Clinical evidence (case-control study) and preclinical evidence (cell model)	Partially conservative	(121)

##### 3.5.2.1 Regulation of cell proliferation, apoptosis and cell cycle progression

Abnormal expression of *PER1* disrupts cell cycle progression, inhibits DNA damage repair, and affects tumor development by regulating cell proliferation and apoptosis. Studies have shown that *PER1* overexpression sensitizes human cancer cells to DNA damage-induced apoptosis, whereas *PER1* inhibition attenuates apoptosis in similarly treated cells (107, 108). Abnormal *PER1* expression leads to dysregulation of multiple genes associated with cell cycle arrest and apoptosis, including Cyclin (CCN) B1, D, E, WEE1, cyclin-dependent kinase (CDK) 1, c-myc, tumor protein p53 (p53), and cyclin-dependent kinase inhibitor 1A (p21) (109–111). As a key regulator of DNA damage repair and cell cycle, p53 mediates *PER1*-dependent transcriptional regulation of WEE1 and CCNB1 (112, 113). In cholangiocarcinoma, *PER1* overexpression suppresses cell proliferation by upregulating cell cycle regulators (e.g., WEE1, CREB phosphatase 1, CRE-BP1), increasing the proportion of G2/M and S phase cells, and reducing G1 phase cell populations (114). In glioma, *PER1* may enhanced checkpoint kinase 2 (CHK2) - p53 signaling and proapoptotic processes and downregulation of *PER1* decreases radiosensitivity and apoptosis in X-ray-irradiated U343 glioma cells (111). *PER1* also regulates G1/S transition by modulating p21-mediated inhibition of CDK2/4/6 (113, 115), and sensitizes cancer cells to ionizing radiation-induced apoptosis via c-Myc-dependent suppression of p21-mediated cell cycle arrest (109). In oral squamous cell carcinoma, KO of *PER1* promotes cell growth, proliferation, apoptosis resistance, migration and invasion, accompanied by upregulated mRNA expression of Ki-67, mouse double minute 2 homolog (MDM2), B-cell lymphoma 2 (BCL-2), MMP2, MMP9, and downregulated expression of cellular MYC proto-oncogene (C-MYC), p53, BCL-2 associated X protein (BAX), tissue inhibitor of metalloproteinases 2 (TIMP-2) (116). Additionally, *PER1* overexpression reduces invasion and migration of nasopharyngeal carcinoma cells (117).

##### 3.5.2.2 Suppression of metabolic reprogramming

In prostate cancer, *PER1* interacts with the androgen receptor (AR), serving as a negative regulator of AR activity. Activated AR stimulates *PER1* expression, which in turn attenuates AR signaling to maintain hormonal homeostasis (101). The methylation status of the *PER1* promoter is negatively correlated with estrogen receptor (ER)-positive expression in breast cancer, suggesting that *PER1* methylation variations may influence ER expression (118). In oral squamous cell carcinoma (OSCC), *PER1* inhibits glycolysis-mediated cell proliferation by forming a PER1/receptor for activated C kinase 1 (RACK1)/phosphatidylinositol 3-kinase (PI3K) complex, regulating PI3K stability, and modulating PI3K/protein kinase B (AKT) signaling-dependent mechanisms (103, 119). In trastuzumab-resistant gastric cancer cells, *PER1* complexes with PPAR-γ to promote upregulation of hexokinase 2 (HK2), thereby enhancing glycolytic activity (120).

##### 3.5.2.3 Modulation of immune microenvironment

*PER1* expression levels are associated with tumor immune infiltration, participating in immune evasion by influencing immune factor expression and immune cell recruitment. Studies have shown that *PER1* expression in ovarian cancer is positively correlated with infiltration of neutrophils, Treg cells, and M2-type macrophages (106). Additionally, *PER1* overexpression in endometrial cancer cells promotes the expression of immune factors TNF-α and IL-6, while upregulating immune checkpoints programmed death-1 (PD-1)/programmed death-ligand 1 (PD-L1). This may inhibit tumor invasion by activating immune responses (121).

## 4 Conclusions and prospects

As a core gene of the circadian clock, *PER1* plays a critical role in the occurrence and development of various diseases through complex mechanisms and signaling pathways (**Table 6**). Its abnormal expression and functional disorders widely affect the physiological and pathological processes of the organism. Based on existing studies, the function of *PER1* exhibits tissue specificity, but its mechanisms of action in different organs/diseases remain incompletely understood. Moreover, most current research is based on animal or cell models, but there are not many clinical studies on *PER1* and large-scale clinical validation remains lacking (**Table 7**), especially in the development of gene therapy or targeted drugs. In-depth studies on the mechanisms of *PER1* in different diseases provide new perspectives for understanding the pathogenesis of these diseases and lay a theoretical foundation for the development of therapeutic strategies based on circadian rhythm regulation.

**Table 6:** *PER1* interactions with core signaling pathways and related pathologies.

Disease field	Core signaling pathway	Interaction between *PER1* and signaling pathway	Related pathological manifestations	Evidence level	Cross-species conservation of mechanism	Reference
Cardiovascular Diseases	ET-1/RAAS	Regulates ET-1 expression and activity, influences renal sodium transport and blood pressure rhythm, antagonizes salt load-induced vascular contraction and fibrosis, and participates in hypertension progression via RAAS regulation	Hypertension, renal injury, vascular fibrosis	Preclinical evidence (animal model)	Partially conservative	(37, 38)
	MR	Maintains close regulatory relationship with MR; CK1-mediated phosphorylation of PER1 indirectly modulates MR function, affecting renal sodium retention and aldosterone levels	Salt-sensitive hypertension, renal sodium metabolism disorder	Preclinical evidence (animal and cell models)	Conservative	(24, 25, 39, 41)
	cAMP/PKA/CREB pathway	Inhibited by GPR183, leading to disrupted circadian rhythm signals and promoted endothelial senescence and dysfunction through this pathway	Hypertension-related endothelial injury, vascular aging	Preclinical evidence (animal model)	Partially conservative	(42)
	SIRT1	Maintains circadian rhythm, activates SIRT1 and other rhythmic genes, enhances myocardial antioxidant and anti-inflammatory capacities to alleviate ischemia-reperfusion injury	Myocardial ischemia-reperfusion injury	Preclinical evidence (animal model)	Conservative pending verification	(45)
	IGF2 anti-inflammatory pathway	Indirectly regulates IGF2-mediated anti-inflammatory pathway by maintaining normal SCN function or synergizing with other rhythmic genes, improving cardiac repair after myocardial infarction	Impaired cardiac repair after myocardial infarction, vascular inflammation	Preclinical evidence (animal model)	Conservative pending verification	(46)
	VEGF pathway	Overexpression inhibits macrophage M1 polarization and proinflammatory cytokine release, activates VEGF pathway to promote blood flow recovery and alleviate limb ischemia injury	Peripheral arterial disease, tissue injury after limb ischemia	Preclinical evidence (animal and cell models)	Partially conservative	(47)
Neurological Disorders	GluA1/p62 autophagy pathway	Maintains p62-mediated selective autophagic degradation of GluA1; *PER1* deficiency disrupts this process, causes GluA1 accumulation and reduced autophagic activity, exacerbating ischemic brain injury	Ischemic brain injury, enlarged cerebral infarct volume	Preclinical evidence (animal model)	Conservative pending verification	(49)
	LC3	Deficiency slows autophagic machinery (reduced basal LC3 levels, unchanged LC3-II/LC3-I ratio), increases neuronal susceptibility to cerebral ischemia	Ischemic brain injury, neuronal damage	Preclinical evidence (animal and cell models)	Partially conservative	(50, 51)
	Cortisol/Melatonin	Abnormal expression causes sleep-wake cycle disorder, increased cortisol, and decreased melatonin secretion, leading to post-stroke sleep disturbance and chronic insomnia	Post-ischemic stroke sleep disorder, chronic insomnia	Preclinical evidence (animal model)	Conservative	(53, 54)
	Dopamine D2 receptor	Upregulation in nucleus accumbens is associated with dopamine D2 receptor activation, affecting neurotransmitter release and participating in PD-related dyskinesia	PD-related dyskinesia	Preclinical evidence (animal model)	Conservative	(60, 61)
Metabolic & Endocrine Diseases	PPARγ/Srebp1c	Overexpression upregulates PPARγ and its target genes, promotes adipogenic gene (Srebp1c) expression and lipid synthesis; PER1 deletion reduces hepatic lipid accumulation and alleviates ethanol-induced liver injury	Abnormal lipid metabolism, hepatic lipid accumulation, non-alcoholic fatty liver	Preclinical evidence (animal model)	Conservative	(70, 71)
	PER1/PKA pathway	Directly interacts with key hepatic bile acid synthesis enzymes (e.g., cholesterol 7α-hydroxylase), participates in rhythmic bile acid biosynthesis via PKA-mediated phosphorylation; fasting/high-fat stress enhances PER1 expression to increase fat absorption	Bile acid metabolism disorder, abnormal fat absorption	Preclinical evidence (animal model)	Conservative pending verification	(72)
	SGLT1	Regulates E-box element activity in SGLT1 promoter, modulates SGLT1 transcription and glucose absorption, affecting glucose homeostasis	Glucose metabolism disorder, impaired blood glucose homeostasis	Preclinical evidence (animal and cell models)	Partially conservative	(74)
	AMPK-SIRT1 pathway	Glucose restriction induces PER1 expression via this pathway; PER1 deficiency exacerbates diet-induced insulin resistance and glucose intolerance	Type 2 diabetes, insulin resistance, diet-induced glucose intolerance	Preclinical evidence (animal model)	Conservative pending verification	(76, 80)
	SREBF2/ALOX15 pathway	Inhibits this pathway to promote granulosa cell ferroptosis and lipid metabolism disorder in PCOS	Polycystic Ovary Syndrome (PCOS), abnormal lipid metabolism	Preclinical evidence (animal model)	Conservative pending verification	(84)
	IGFBP4/SHBG	Decreased PER1/PER2 expression promotes androgen excess via regulating IGFBP4 and SHBG in the liver	PCOS, hyperandrogenism	Clinical evidence (case-control study) + Preclinical evidence (animal and cell models)	Conservative	(85)
	Atox1/Atp7b	Overexpression upregulates Atox1 to reduce intracellular copper accumulation and alleviate nephrotoxicity; overexpression may exacerbate Cu-induced hepatotoxicity by downregulating Atp7b in hepatocytes	Copper-induced nephrotoxicity, Cu-induced hepatic toxicity	Preclinical evidence (animal and cell models)	Conservative pending verification	(86, 87)
Immune & Inflammatory Diseases	mTORC1 pathway	Regulated by adrenergic and glucocorticoid stress signaling; PER1 expression inhibits mTORC1 activity and suppresses Th1 polarization to maintain immune homeostasis	Autoimmune diseases, T-cell subset imbalance	Preclinical evidence (cell model)	Conservative pending verification	(88)
	Ferroptosis pathway	PER1/PER2 double knockout induces ferroptosis in splenic lymphocytes, leading to reduced immune cell counts, lymphoid tissue structural damage, and impaired immune function	Immune deficiency, lymphoid tissue structural damage	Preclinical evidence (animal model)	Conservative pending verification	(89)
	COX7C	Maintains Treg cell stability and function via COX7C-dependent mitochondrial metabolism; decreased PER1 expression impairs Treg immunosuppressive function	Autoimmune uveitis	Clinical evidence (case-control study) + Preclinical evidence (animal and cell models)	Conservative	(90)
	NF-κB pathway	Regulates IL-1β-induced MMP13 expression in mandibular condylar chondrocytes; activates NF-κB pathway to promote CCL2/IL-6 production in spinal astrocytes	Temporomandibular joint osteoarthritis, spinal inflammation, intestinal barrier injury	Preclinical evidence (animal and cell models)	Conservative pending verification	(95, 96, 98)
	PERLD1/sGPI-AP	PERLD1 haplotype alters PBMC sensitivity by influencing sGPI-AP levels, contributing to individual susceptibility to allergic asthma	Allergic asthma	Clinical evidence (case-control study)	Conservative	(100)
Tumors	p53/WEE1/CCNB1 pathway	Regulates p53-mediated transcription of WEE1 and CCNB1, affects cell cycle G2/M progression and DNA damage repair, enhances cancer cell sensitivity to DNA damage-induced apoptosis	Cholangiocarcinoma, glioma, multiple tumor proliferation, radiotherapy resistance	Preclinical evidence (animal and cell models)	Conservative	(109, 111, 114)
	PER1/RACK1/PI3K pathway	Forms complex with RACK1 to regulate PI3K stability, inhibits PI3K/AKT-mediated glycolysis, suppresses proliferation and invasion of oral squamous cell carcinoma (OSCC)	OSCC proliferation and invasion	Preclinical evidence (animal and cell models)	Conservative	(103, 119)
	AR	Interacts with androgen receptor (AR) as a negative regulator of AR activity, maintains hormonal homeostasis, inhibits prostate cancer progression	Prostate cancer progression	Preclinical evidence (animal and cell models)	Conservative	(101)
	PPAR-γ/HK2	Forms complex with PPAR-γ to promote HK2 upregulation, enhances glycolytic activity, participates in the development of trastuzumab-resistant gastric cancer	Trastuzumab-resistant gastric cancer, tumor metabolic reprogramming	Preclinical evidence (animal and cell models)	Conservative	(120)
	PD-1/PD-L1	Overexpression promotes TNF-α/IL-6 expression and PD-1/PD-L1 upregulation, activates anti-tumor immune responses, inhibits endometrial cancer invasion and immune escape	Endometrial cancer invasion, tumor immune escape	Clinical evidence (case-control study) + Preclinical evidence (cell model)	Partially conservative	(121)

**Table 7:** Clinical studies on *PER1*’s role in various diseases.

Types of diseases	Related signaling molecules/pathways	Specific functions/mechanisms	Evidence level	Cross-species conservation of mechanism	References
PD	*PER1* gene variants (e.g.,loss-of-function, rs2225380 variants)	Loss-of-function variants of *PER1* are associated with motor dysfunctions in PD, such as dyskinesia	IIb	Conservative pending verification	(58)
		The rs2225380 variant is positively correlated with PIGD subtype of sporadic PD	IIIa		(59)
AD	*PER1* gene polymorphism (rs3027178)	The G allele of rs3027178 confers a protective effect against AD	IIIa	Conservative pending verification	(63)
PCOS	IGFBP4, SHBG	Decreased *PER1* and *PER2* expression can result in excessive androgen production	IIIa	Conservative	(85)
Uveitis	COX7C	High Tregs of COX7C were positively correlated with the severity of uveitis	IIIa	Conservative	(90)
IBD	–	*PER1* expression was significantly downregulated in intestinal tissues and PBMCs in IBD patients	IIb	Conservative	(97)
Allergic asthma	sGPI-AP	PERLD1 haplotype alters the sensitivity of PBMCs through influencing sGPI-AP levels,	IIIa	Conservative	
OSCC	–	Per1 expression is significantly downregulated in OSCC	IIIa	Conservative	(103)

Future research on *PER1* is expected to achieve breakthroughs in the following aspects: First, to further clarify the specific regulatory mechanisms of *PER1* in different tissues and cell types, particularly its dynamic changes during disease occurrence and progression. Second, to develop drugs or therapeutic approaches that can precisely target *PER1* and its related signaling pathways, such as the development of drugs based on CK1 inhibitors or FMRP mimetics, to restore the normal function and rhythm of *PER1*. Third, to explore *PER1* as a biomarker for disease diagnosis and prognosis assessment, enabling early disease diagnosis and personalized treatment by detecting *PER1* expression levels, rhythm changes, etc. With the continuous in-depth study of *PER1*, it is believed that disease prevention and treatment strategies based on circadian rhythm regulation will bring new hope for improving human health.

## Glossary

PER: period
BMAL1: brain and muscle arnt-like 1
CLOCK: circadian locomotor output cycles kaput
CRY: cryptochrome
SCN: suprachiasmatic nucleus
TTFL: transcription-translation feedback loop
RORs: RAR-related orphan receptors
NR1D1: nuclear receptor subfamily 1 group D member 1
mRNA: messenger RNA
ZT: Zeitgeber time
CK1: casein kinase 1
FMRP: fragile X mental retardation protein
GR: glucocorticoid receptor
MR: mineralocorticoid receptor
KO: knockout
ET-1: endothelin-1
RAAS: renin-angiotensin-aldosterone system
cAMP: cyclic 3’,5’-adenosine monophosphate
PKA: protein kinase A
CREB: cAMP-response element binding protein
GC: glucocorticoid
SIRT1: sirtuin 1
IGF2: insulin-like growth factor 2
HLI: hindlimb ischemia
VEGF: vascular endothelial growth factor
GluA1: glutamate ionotropic receptor AMPA type subunit 1
p62: sequestosome 1
Cyt c: cytochrome c
Apaf1: apoptotic protease activating factor 1
Rus: ruscogenin
Nrf2/HO-1: nuclear factor erythroid 2-related factor 2/heme oxygenase-1
PD: Parkinson’s disease
PIGD: postural instability and gait disorder
AD: Alzheimer’s disease
*CHI3L1*: chitinase-3-like protein 1
Aβ: amyloid-β
SYT14: synaptotagmin 14
PPAR-γ: peroxisome proliferator-activated receptor - gamma
SGLT1: sodium-glucose cotransporter 1
AMPK: AMP-activated protein kinase
PCOS: polycystic ovary syndrome
IGFBP4: insulin-like growth factor-binding protein 4
SHBG: sex hormone binding globulin
SREBF2: sterol regulatory element-binding factor 2
ALOX15: arachidonate 15-lipoxygenase
Atox1: Antioxidant 1 Copper Chaperone
Atp7b: ATPase copper transporting beta
Treg: regulatory T
COX7C: cytochrome c oxidase subunit 7C
NK: natural killer
IFN-γ: interferon-gamma
TNF-α: tumor necrosis factor - alpha
IL: interleukin
CCL2: chemokine (C-C motif) ligand 2
Ccr2: CC chemokine receptor 2
MMP: matrix metalloproteinase
NF-κB: nuclear factor-kappa B
p38: p38 mitogen-activated protein kinase
JNK1: c-Jun N-terminal kinase 1
IBD: inflammatory bowel disease
WEE1: wee1-like protein kinase
cIAP2: cellular inhibitor of apoptosis protein-2
PERLD1: PER1-like domain-containing protein 1
PBMCs: peripheral blood mononuclear cells
sGPI-AP: soluble glycosylphosphatidylinositol anchor protein
CCN: Cyclin
CDK: cyclin-dependent kinase
p53: tumor protein p53
p21: cyclin-dependent kinase inhibitor 1A
CHK2: checkpoint kinase 2
MDM2: mouse double minute 2 homolog
BCL-2: B-cell lymphoma 2
C-MYC: cellular MYC proto-oncogene
BAX: BCL-2 associated X protein
TIMP-2: tissue inhibitor of metalloproteinases 2
AR: androgen receptor
ER: estrogen receptor
OSCC: oral squamous cell carcinoma
RACK1: receptor for activated C kinase 1
PI3K: phosphatidylinositol 3-kinase
AKT: protein kinase B
HK2: hexokinase 2
PD-1: programmed death-1
PD-L1: programmed death-ligand 1

## References

[1] Richards J Gumz ML . Advances in understanding the Peripheral circadian Clocks. FASEB J. (2012) 26:3602–13. doi: 10.1096/fj.12-203554, PMID: 22661008 PMC3425819

[2] Lee Y Field JM Sehgal A . Circadian rhythms, disease and chronotherapy. J Biol rhythms. (2021) 36:503–31. doi: 10.1177/07487304211044301, PMID: 34547953 PMC9197224

[3] Patke A Young MW Axelrod S . Molecular mechanisms and physiological importance of circadian rhythms. Nat Rev Mol Cell Biol. (2020) 21:67–84. doi: 10.1038/s41580-019-0179-2, PMID: 31768006

[4] Wang XL Li L . Circadian clock regulates inflammation and the development of neurodegeneration. Front Cell Infection Microbiol. (2021) 11:696554. doi: 10.3389/fcimb.2021.696554, PMID: 34595127 PMC8476957

[5] Riede SJ van der Vinne V Hut RA . The flexible Clock: predictive and reactive homeostasis, energy balance and the circadian regulation of sleep-wake timing. J Exp Biol. (2017) 220:738–49. doi: 10.1242/jeb.130757, PMID: 28250173

[6] Aoyama S Shibata S . Time-of-day-dependent physiological responses to meal and exercise. Front Nutr. (2020) 7:18. doi: 10.3389/fnut.2020.00018, PMID: 32181258 PMC7059348

[7] Tomatsu S Abbott SM Attarian H . Clinical chronobiology: circadian rhythms in health and disease. Semin Neurol. (2025) 45:317–32. doi: 10.1055/a-2538-3259, PMID: 39961369 PMC12323393

[8] Catalano F De Vito F Cassano V Fiorentino TV Sciacqua A Hribal ML Circadian clock desynchronization and insulin resistance. Int J Environ Res Public Health. (2022) 20:29. doi: 10.3390/ijerph20010029, PMID: 36612350 PMC9819930

[9] Reppert SM Weaver DR . Molecular analysis of mammalian circadian rhythms. Annu Rev Physiol. (2001) 63:647–76. doi: 10.1146/annurev.physiol.63.1.647, PMID: 11181971

[10] Jones JR Mcmahon DG . The core Clock gene Per1 phases molecular and electrical circadian rhythms in SCN neurons. PeerJ. (2016) 4:e2297. doi: 10.7717/peerj.2297, PMID: 27602274 PMC4991845

[11] Liu S Chen B Cai Y-N Wen M Zuo X-H Human Perl gene expression in Peripheral mononuclearsystem in young adults. Chin J Modern Med. (2006) 16:381–383,386.

[12] Cao X Wang L Selby CP Lindsey-Boltz LA Sancar A Analysis of mammalian circadian Clock protein complexes over a circadian cycle. J Biol Chem. (2023) 299:102929. doi: 10.1016/j.jbc.2023.102929, PMID: 36682495 PMC9950529

[13] Otobe Y Jeong EM Ito S Shinohara Y Kurabayashi N Aiba A . Phosphorylation of DNA-binding domains of Clock–Bmal1 complex for Per-dependent inhibition in circadian Clock of mammalian cells. Proc Natl Acad Sci. (2024) 121:e2316858121. doi: 10.1073/pnas.2316858121, PMID: 38805270 PMC11161756

[14] Reppert SM Weaver DR . Coordination of circadian timing in mammals. Nature. (2002) 418:935–41. doi: 10.1038/nature00965, PMID: 12198538

[15] Curtis AM Bellet MM Sassone-Corsi P O'Neill LA Circadian Clock proteins and immunity. Immunity. (2014) 40:178–86. doi: 10.1016/j.immuni.2014.02.002, PMID: 24560196

[16] Man K Loudon A Chawla A . Immunity around the clock. Sci (New York N.Y.). (2016) 354:999–1003. doi: 10.1126/science.aah4966, PMID: 27885005 PMC5247264

[17] Kwak JS . Functional and regulatory diversification of Period genes responsible for circadian rhythm in vertebrates. G3 (Bethesda). (2024) 14:jkae162. doi: 10.1093/g3journal/jkae162, PMID: 39028850 PMC11457068

[18] Bae K Jin X Maywood ES Hastings MH Reppert SM Weaver DR Differential functions of mPer1, mPer2, and mPer3 in the SCN circadian Clock. Neuron. (2001) 30:525–36. doi: 10.1016/S0896-6273(01)00302-6, PMID: 11395012

[19] Bernstein R Gaddameedhi S . Time is running out: the circadian clock suggests sex and aging differences in human epidermis. J Invest Dermatol. (2024) 144:931–4. doi: 10.1016/j.jid.2023.12.026, PMID: 38493382

[20] Fahrenkrug J Georg B Hannibal J Hindersson P Gräs S Diurnal rhythmicity of the Clock genes Per1 and Per2 in the rat ovary. Endocrinology. (2006) 147:3769–76. doi: 10.1210/en.2006-0305, PMID: 16675517

[21] Nakamaru E Seki K Shirahata Y Adachi M Sakabe N Matsuo T . Periodic expression of Per1 gene is restored in chipmunk liver during interbout arousal in mammalian hibernation. Sci Rep. (2025) 15:4403. doi: 10.1038/s41598-025-87299-8, PMID: 39948130 PMC11825846

[22] Sato RY Yamanaka Y . Nonphotic entrainment of central and Peripheral circadian Clocks in mice by scheduled voluntary exercise under constant darkness. Am J Physiol Regulatory Integr Comp Physiol. (2023) 324:R526–35. doi: 10.1152/ajpregu.00320.2022, PMID: 36802951

[23] Cermakian N . Altered behavioral rhythms and Clock gene expression in mice with a targeted mutation in the Period1 gene. EMBO J. (2001) 20:3967–74. doi: 10.1093/emboj/20.15.3967, PMID: 11483500 PMC149149

[24] Philpott JM Freeberg AM Park J Lee K Ricci CG Hunt SR . Period phosphorylation leads to feedback inhibition of CK1 activity to control circadian Period. Mol Cell. (2023) 83:1677–1692.e8. doi: 10.1016/j.molcel.2023.04.019, PMID: 37207626 PMC11684667

[25] Park J Lee K Kim H Shin H Lee C Endogenous circadian reporters reveal functional differences of PerIOD paralogs and the significance of PerIOD: CK1 stable interaction. Proc Natl Acad Sci United States America. (2023) 120:e2212255120. doi: 10.1073/pnas.2212255120, PMID: 36724252 PMC9962996

[26] Kim TJ Sung JH Shin JC Kim DY CRISPR/Cas-mediated Fubp1 silencing disrupts circadian oscillation of Per1 protein via downregulating Syncrip expression. Cell Biol Int. (2020) 44:424–32. doi: 10.1002/cbin.11242, PMID: 31535751

[27] Tang X Zhang J Li X Hu Y Liu D Li JD . FMRP binds Per1 mRNA and downregulates its protein expression in mice. Mol Brain. (2023) 16:33. doi: 10.1186/s13041-023-01023-z, PMID: 37020302 PMC10077598

[28] Sun J Zhang Y Adams JA Higgins CB Kelly SC Zhang H . Hepatocyte Period 1 dictates oxidative substrate selection independent of the core circadian Clock. Cell Rep. (2024) 43:114865. doi: 10.1016/j.celrep.2024.114865, PMID: 39412985 PMC11601098

[29] Xu L Wu T Li H Ni Y Fu Z An individual 12-h shift of the light-dark cycle alters the pancreatic and duodenal circadian rhythm and digestive function. Acta Biochim Et Biophys Sin. (2017) 49:954–61. doi: 10.1093/abbs/gmx084, PMID: 28981604

[30] Rigamonti AE Bollati V Favero C Albetti B Caroli D De Col A . Changes in DNA methylation of clock genes in obese adolescents after a short-term body weight reduction program: A possible metabolic and endocrine chrono-resynchronization. Int J Environ Res Public Health. (2022) 19:15492. doi: 10.3390/ijerph192315492, PMID: 36497566 PMC9738941

[31] Pivovarova O Jürchott K Rudovich N Hornemann S Ye L Möckel S . Changes of dietary fat and carbohydrate content alter central and peripheral clock in humans. J Clin Endocrinol Metab. (2015) 100:2291–302. doi: 10.1210/jc.2014-3868, PMID: 25822100

[32] Le Billan F Amazit L Bleakley K Xue QY Pussard E Lhadj C . Corticosteroid receptors adopt distinct cyclical transcriptional signatures. FASEB journal: Off Publ Fed Am Societies Exp Biol. (2018) 32:5626–39. doi: 10.1096/fj.201800391RR, PMID: 29733691

[33] Saracino PG Rossetti ML Steiner JL Gordon BS . Hormonal regulation of core Clock gene expression in skeletal muscle following acute aerobic exercise. Biochem Biophys Res Commun. (2019) 508:871–6. doi: 10.1016/j.bbrc.2018.12.034, PMID: 30538043

[34] Knoedler JR Sáenz de Miera C Subramani A Denver RJ . An Intact Krüppel-like factor 9 Gene Is Required for Acute Liver Period 1 mRNA Response to Restraint Stress. Endocrinology. (2021) 162:bqab083. doi: 10.1210/endocr/bqab083, PMID: 33904929 PMC8312639

[35] Lother A Jaisser F Wenzel UO . Emerging fields for therapeutic targeting of the aldosterone–mineralocorticoid receptor signaling pathway. Br J Pharmacol. (2022) 179:3099–102. doi: 10.1111/bph.15808, PMID: 35174485

[36] Wu Q Ren QY Wang X Bai HY Tian DD Gao GF . Cellular iron depletion enhances behavioral rhythm by limiting brain Per1 expression in mice. CNS Neurosci Ther. (2024) 30:e14592. doi: 10.1111/cns.14592, PMID: 38385622 PMC10883092

[37] Costello HM Juffre A Cheng KY Bratanatawira P Crislip GR Zietara A . The circadian clock protein per1 is important in maintaining endothelin axis regulation in dahl salt sensitive rats. Can J Physiol Pharmacol. (2023) 101:136–46. doi: 10.1139/cjpp-2022-0134, PMID: 36450128 PMC9992312

[38] Zietara A Spires DR Juffre A Costello HM Crislip GR Douma LG . Knockout of the circadian Clock protein Per1 exacerbates hyPertension and increases kidney injury in Dahl salt-sensitive rats. HyPertension (Dallas Tex. : 1979). (2022) 79:2519–29. doi: 10.1161/HYPERTENSIONAHA.122.19316, PMID: 36093781 PMC9669134

[39] Douma LG Costello HM Crislip GR Cheng KY Lynch IJ Juffre A . Kidney-specific KO of the circadian Clock protein Per1 alters renal Na+ handling, aldosterone levels, and kidney/adrenal gene expression. Am J Physiol - Renal Physiol. (2022) 322:F449–59. doi: 10.1152/ajprenal.00385.2021, PMID: 35129370 PMC9169971

[40] Douma LG Crislip GR Cheng KY Barral D Masten S Holzworth M . Knockout of the circadian clock protein per1 results in sex-dependent alterations of ET-1 production in mice in response to a high salt diet plus mineralocorticoid treatment. Can J Physiol Pharmacol. (2020) 98:579–86. doi: 10.1139/cjpp-2019-0688, PMID: 32437627 PMC7605171

[41] Ruhs S Griesler B Huebschmann R Stroedecke K Straetz N Ihling C . Modulation of transcriptional mineralocorticoid receptor activity by casein kinase 1. FASEB J. (2022) 36:e22059. doi: 10.1096/fj.202100977RR, PMID: 34847273

[42] Chu Q Li YJ Wu J Gao Y Guo X Li J . Oxysterol sensing through GPR183 triggers endothelial senescence in hyPertension. Circ Res. (2024) 135:708–21. doi: 10.1161/CIRCRESAHA.124.324722, PMID: 39176657

[43] Ginty AT Kraynak TE Fisher JP Gianaros PJ . Cardiovascular and autonomic reactivity to psychological stress: Neurophysiological substrates and links to cardiovascular disease. Autonomic Neuroscience: Basic Clin. (2017) 207:2–9. doi: 10.1016/j.autneu.2017.03.003, PMID: 28391987 PMC5600671

[44] van Alem CM Boonstra M Prins J Bezhaeva T van Essen MFR Ruben JM . Local delivery of liposomal prednisolone leads to an anti-inflammatory profile in renal ischaemia-rePerfusion injury in the rat. Nephrology Dialysis Transplantation: Off Publ Eur Dialysis Transplant Assoc - Eur Renal Assoc. (2018) 33:44–53. doi: 10.1093/ndt/gfx204, PMID: 28992069

[45] Zhang B Wang C Guo M Zhu F Yu Z Zhang W . Circadian rhythm-dependent therapy by composite targeted polyphenol nanoparticles for myocardial ischemia-rePerfusion injury. ACS nano. (2024) 18:28154–69. doi: 10.1021/acsnano.4c07690, PMID: 39373010

[46] Hao K-L Zhai Q-C Gu Y Chen Y-Q Wang Y-N Liu R . Disturbance of suprachiasmatic nucleus function improves cardiac repair after myocardial infarction by IGF2-mediated macrophage transition. . Acta Pharmacologica Sin. (2023) 44:1612–24. doi: 10.1038/s41401-023-01059-w, PMID: 36747104 PMC10374569

[47] Ding Y Wan S Ma L Wei K Ye K . Per1 promotes functional recovery of mice with hindlimb ischemia by inducing anti-inflammatory macrophage polarization. Biochem Biophys Res Commun. (2023) 644:62–9. doi: 10.1016/j.bbrc.2023.01.001, PMID: 36634583

[48] He M Li L Li J Chen S Shi H . rs2253820 variant controls blood pressure dip after stroke by increasing clock-bmal1 expression. Trans Stroke Res. (2023) 14:472–89. doi: 10.1007/s12975-022-01063-y, PMID: 35870088

[49] Lu H Wang Y Fan H Wang Y Fan S Hu S . GluA1 degradation by autophagy contributes to circadian rhythm effects on cerebral ischemia injury. J Neurosci. (2023) 43:2381–97. doi: 10.1523/JNEUROSCI.1914-22.2023, PMID: 36813576 PMC10072305

[50] Wiebking N Maronde E Rami AH . Increased neuronal injury in clock gene Per-1 deficient-mice after cerebral ischemia. Curr Neurovasc Res. (2013) 10:112–25. doi: 10.2174/1567202611310020004, PMID: 23469952

[51] Rami A Fekadu J Rawashdeh O . The hippocampal autophagic machinery is depressed in the absence of the circadian clock protein per1 that may lead to vulnerability during cerebral ischemia. Curr Neurovascular Res. (2017) 14:207–14. doi: 10.2174/1567202614666170619083239, PMID: 28625127

[52] Sochal M Ditmer M Tarasiuk-Zawadzka A Binienda A Turkiewicz S Wysokiński A . Circadian rhythm genes and their association with sleep and sleep restriction. Int J Mol Sci. (2024) 25:10445. doi: 10.3390/ijms251910445, PMID: 39408776 PMC11476465

[53] Kopp C Albrecht U Zheng B Tobler I . Homeostatic sleep regulation is preserved in mPer1 and mPer2 mutant mice. Eur J Neurosci. (2002) 16:1099–106. doi: 10.1046/j.1460-9568.2002.02156.x, PMID: 12383239

[54] Chu TT Sun C Zheng YH Gao WY Zhao LL Zhang JY . Study on the mechanisms of ischemic stroke impacting sleep homeostasis and circadian rhythms in rats. CNS Neurosci Ther. (2025) 31:e70153. doi: 10.1111/cns.70153, PMID: 39957482 PMC11831068

[55] Emeklİ R İsmaİloğullari S Bayram A Akalin H Tuncel G Dündar M . Comparing expression levels of PerIOD genes Per1, Per2 and Per3 in chronic insomnia patients and medical staff working in the night shift. Sleep Med. (2020) 73:101–5. doi: 10.1016/j.sleep.2020.04.027, PMID: 32805476

[56] Hu X Li J Wang X Liu H Wang T Lin Z . Neuroprotective effect of melatonin on sleep disorders associated with parkinson’s disease. Antioxidants. (2023) 12:396. doi: 10.3390/antiox12020396, PMID: 36829955 PMC9952101

[57] Li T Cheng C Jia C Leng Y Qian J Yu H . Peripheral clock system abnormalities in patients with parkinson’s disease. Front Aging Neurosci. (2021) 13:736026. doi: 10.3389/fnagi.2021.736026, PMID: 34658839 PMC8519399

[58] Xiang Y Huang JJ Wang Y Huang XR Zeng Q Li L . Evaluating the genetic role of circadian clock genes in parkinson’s disease. Mol Neurobiol. (2023) 60:2729–36. doi: 10.1007/s12035-023-03243-9, PMID: 36717479

[59] Gu Z Wang BB Zhang YB Ding H Zhang Y Yu J . Association of ARNTL and Per1 genes with Parkinson’s disease: a case-control study of Han Chinese. Sci Rep. (2015) 5:15891. doi: 10.1038/srep15891, PMID: 26507264 PMC4623766

[60] Sharma R Parikh M Mishra V Sahota P Thakkar M . Activation of dopamine D2 receptors in the medial shell region of the nucleus accumbens increases Per1 expression to enhance alcohol consumption. Addict Biol. (2022) 27:e13133. doi: 10.1111/adb.13133, PMID: 35032086

[61] Olejniczak I Ripperger JA Sandrelli F Schnell A Mansencal-Strittmatter L Wendrich K . Light affects behavioral despair involving the Clock gene Period 1. . PloS Genet. (2021) 17:e1009625. doi: 10.1371/journal.pgen.1009625, PMID: 34237069 PMC8266116

[62] Zhao R Sun JB Deng H Cheng C Li X Wang FM . Per1 gene polymorphisms influence the relationship between brain white matter microstructure and depression risk. Front Psychiatry. (2022) 13:1022442. doi: 10.3389/fpsyt.2022.1022442, PMID: 36440417 PMC9691780

[63] Bacalini MG Palombo F Garagnani P Giuliani C Fiorini C Caporali L . Association of rs3027178 polymorphism in the circadian Clock gene Per1 with susceptibility to Alzheimer’s disease and longevity in an Italian population. GeroScience. (2021) 44:881–96. doi: 10.1007/s11357-021-00477-0, PMID: 34921659 PMC9135916

[64] Lananna BV McKee CA King MW Del-Aguila JL Dimitry JM Farias FHG . Chi3l1/YKL-40 is controlled by the astrocyte circadian Clock and regulates neuroinflammation and Alzheimer Disease pathogenesis. Sci Trans Med. (2020) 12:eaax3519. doi: 10.1126/scitranslmed.aax3519, PMID: 33328329 PMC7808313

[65] Börner JH Rawashdeh O Rami A . Exacerbated age-related hippocampal alterations of microglia morphology, β-amyloid and lipofuscin deposition and presenilin overexpression in per1–/—Mice. Antioxidants. (2021) 10:1330. doi: 10.3390/antiox10091330, PMID: 34572962 PMC8469021

[66] Rawashdeh O Jilg A Maronde E Fahrenkrug J Stehle JH . Period1 gates the circadian modulation of memory-relevant signaling in mouse hippocampus by regulating the nuclear shuttling of the CREB kinase pP90RSK. J Neurochemistry. (2016) 138:731–45. doi: 10.1111/jnc.13689, PMID: 27246400

[67] Rawashdeh O Jilg A Jedlicka P Slawska J Thomas L Saade A . PERIOD1 coordinates hippocampal rhythms and memory processing with daytime. Hippocampus. (2014) 24:712–23. doi: 10.1002/hipo.22262, PMID: 24550127

[68] Zhang Y Zhang C-Y Yuan J Jiang H Sun P Hui L . Human mood disorder risk gene Synaptotagmin-14 contributes to mania-like behaviors in mice. Mol Psychiatry. (2025) 30:3466–77. doi: 10.1038/s41380-025-02933-1, PMID: 39966626

[69] Shaito A Al-Mansoob M Ahmad SMS Haider MZ Eid AH Posadino AM . Resveratrol-mediated regulation of mitochondria biogenesis-associated pathways in neurodegenerative diseases: molecular insights and potential therapeutic applications. Curr Neuropharmacology. (2023) 21:1184–201. doi: 10.2174/1570159X20666221012122855, PMID: 36237161 PMC10286596

[70] Wang T Yang P Zhan Y Xia L Hua Z Zhang J . Deletion of circadian gene Per1 alleviates acute ethanol-induced hepatotoxicity in mice. Toxicology. (2013) 314:193–201. doi: 10.1016/j.tox.2013.09.009, PMID: 24144995

[71] Liu CZ Zhou B Meng MY Zhao WJ Wang DM Yuan YW . FOXA3 induction under endoplasmic reticulum stress contributes to non-alcoholic fatty liver disease. J Hepatol. (2021) 75:150–62. doi: 10.1016/j.jhep.2021.01.042, PMID: 33548387

[72] Ge W Sun Q Yang Y Ding Z Liu J Zhang J . Circadian Per1 controls daily fat absorption with the regulation of Per1-PKA on phosphorylation of bile acid synthetase. J Lipid Res. (2023) 64:100390. doi: 10.1016/j.jlr.2023.100390, PMID: 37209828 PMC10276160

[73] Yin J Li Y Han H Ma J Liu G Wu X . Administration of exogenous melatonin improves the diurnal rhythms of the gut microbiota in mice fed a high-fat diet. mSystems. (2020) 5:e00002–20. doi: 10.1128/msystems.00002-20, PMID: 32430404 PMC7253360

[74] Balakrishnan A . Micromanaging the gut: unravelling the regulatory pathways that mediate the intestinal adaptive response. Ann R Coll Surgeons Engl. (2018) 100:165–71. doi: 10.1308/rcsann.2017.0174, PMID: 29364022 PMC5930084

[75] Yamaoka M Maeda N Takayama Y Sekimoto R Tsushima Y Matsuda K . Adipose hypothermia in obesity and its association with period homolog 1, insulin sensitivity, and inflammation in fat. PloS One. (2014) 9:e112813. doi: 10.1371/journal.pone.0112813, PMID: 25397888 PMC4232416

[76] Xu H Li H Woo SL Kim SM Shende VR Neuendorff N . Myeloid cell-specific disruption of period1 and period2 exacerbates diet-induced inflammation and insulin resistance. J Biol Chem. (2014) 289:16374–88. doi: 10.1074/jbc.M113.539601, PMID: 24770415 PMC4047405

[77] Figueroa AL Figueiredo H Rebuffat SA Vieira E Gomis R . Taurine treatment modulates circadian rhythms in mice fed A high fat diet. Sci Rep. (2016) 6:36801. doi: 10.1038/srep36801, PMID: 27857215 PMC5114685

[78] Chen MY Zhang Y Zeng S Li DY You MY Zhang MY . CD36 regulates diurnal glucose metabolism and hepatic Clock to maintain glucose homeostasis in mice. iScience. (2023) 26:106524. doi: 10.1016/j.isci.2023.106524, PMID: 37123238 PMC10139992

[79] Kanou H Nagasawa K Ishii Y Chishima A Hayashi J Haga S . Period1 gene expression in the olfactory bulb and liver of freely moving streptozotocin-treated diabetic mouse. Biochem Biophys Res Commun. (2021) 560:14–20. doi: 10.1016/j.bbrc.2021.04.049, PMID: 33965785

[80] Li B Chen Q Feng Y Wei T Zhong Y Zhang Y . Glucose restriction induces AMPK-SIRT1-mediated circadian Clock gene Per expression and delays NSCLC progression. Cancer Lett. (2023) 576:216424. doi: 10.1016/j.canlet.2023.216424, PMID: 37778683

[81] Liu Z Huang M Wu X Shi G Xing L Dong Z . Per1 phosphorylation specifies feeding rhythm in mice. Cell Rep. (2014) 7:1509–20. doi: 10.1016/j.celrep.2014.04.032, PMID: 24857656

[82] Yan L Rust BM Palmer DG . Time-restricted feeding restores metabolic flexibility in adult mice with excess adiposity. Front Nutr. (2024) 11:1340735. doi: 10.3389/fnut.2024.1340735, PMID: 38425486 PMC10902009

[83] Cui Y Li S Yin Y Li X Li X . Daytime restricted feeding promotes circadian desynchrony and metabolic disruption with changes in bile acids profiles and gut microbiota in C57BL/6 Male Mice. J Nutr Biochem. (2022) 109:109121. doi: 10.1016/j.jnutbio.2022.109121, PMID: 35940511

[84] Chen Y Liu Z Chen H Wen Y Fan L Luo M . Rhythm gene Per1 mediates ferroptosis and lipid metabolism through SREBF2/ALOX15 axis in polycystic ovary syndrome. Biochim Et Biophys Acta Mol Basis Dis. (2024) 1870:167182. doi: 10.1016/j.bbadis.2024.167182, PMID: 38653359

[85] Li S Zhai J Chu W Geng X Chen ZJ Du Y . Altered circadian Clock as a novel therapeutic target for constant darkness-induced insulin resistance and hyPerandrogenism of polycystic ovary syndrome. Trans Research: J Lab Clin Med. (2020) 219:13–29. doi: 10.1016/j.trsl.2020.02.003, PMID: 32119846

[86] Tominaga S Yoshioka H Yokota S Tsukiboshi Y Suzui M Nagai M . CopPer-induced renal toxicity controlled by Period1 through modulation of Atox1 in mice. Biomed Res. (2024) 45:143–9. doi: 10.2220/biomedres.45.143, PMID: 39010190

[87] Tominaga S Yoshioka H Yokota S Tsukiboshi Y Suzui M Nagai M . CopPer-induced diurnal hepatic toxicity is associated with Cry2 and Per1 in mice. Environ Health Prev Med. (2023) 28:78. doi: 10.1265/ehpm.23-00205, PMID: 38092388 PMC10739358

[88] Capelle CM Chen A Zeng N Baron A Grzyb K Arns T . Stress hormone signalling inhibits Th1 polarization in a CD4 T-cell-intrinsic manner via mTORC1 and the circadian gene Per1. Immunology. (2022) 165:428–44. doi: 10.1111/imm.13448, PMID: 35143696 PMC9426625

[89] He R Zhang S Yu J Yu X Wang J Qiu Y . Per1/Per2 knockout Affects Spleen Immune Function in Elderly Mice via Inducing Spleen Lymphocyte Ferroptosis. Int J Mol Sci. (2022) 23:12962. doi: 10.3390/ijms232112962, PMID: 36361751 PMC9657961

[90] Zi S-F Wu X-J Tang Y Liang Y-P Liu X Wang L . Endothelial cell-derived extracellular vesicles promote aberrant neutrophil trafficking and subsequent remote lung injury. Advanced Sci. (2024) 11:e2400647. doi: 10.1002/advs.202400647, PMID: 39119837 PMC11481253

[91] Logan RW Wynne O Levitt D Price D Sarkar DK Altered circadian expression of cytokines and cytolytic factors in splenic natural killer cells of per1–/– mutant mice. J Interferon Cytokine Res. (2013) 33:108–14. doi: 10.1089/jir.2012.0092, PMID: 23402528 PMC3595954

[92] Wang T Wang Z Yang P Xia L Zhou M Wang S . Per1 prevents excessive innate immune response during endotoxin-induced liver injury through regulation of macrophage recruitment in mice. Cell Death Dis. (2016) 7:e2176. doi: 10.1038/cddis.2016.9, PMID: 27054331 PMC4855679

[93] Katoku-Kikyo N Lim S Yuan C Koroth J Nakagawa Y Bradley EW . The circadian regulator Per1 promotes cell reprogramming by inhibiting inflammatory signaling from macrophages. PloS Biol. (2023) 21:e3002419. doi: 10.1371/journal.pbio.3002419, PMID: 38048364 PMC10721173

[94] Hemmers S Rudensky AY . The cell-intrinsic circadian clock is dispensable for lymphocyte differentiation and function. Cell Rep. (2015) 11:1339–49. doi: 10.1016/j.celrep.2015.04.058, PMID: 26004187 PMC4464971

[95] Wei JM Tu SQ Wang YX Zhang S Feng Y Ai H . Clock gene Per1 regulates rat temporomandibular osteoarthritis through NF-κB pathway: an *in vitro* and *in vivo* study. J Orthopaedic Surg Res. (2023) 18:817. doi: 10.1186/s13018-023-04301-7, PMID: 37907921 PMC10619284

[96] Sugimoto T Morioka N Zhang FF Sato K Abe H Hisaoka-Nakashima K . Clock gene Per1 regulates the production of CCL2 and interleukin-6 through p38, JNK1 and NF-κB activation in spinal astrocytes. Mol Cell Neurosci. (2014) 59:37–46. doi: 10.1016/j.mcn.2014.01.003, PMID: 24447840

[97] Weintraub Y Cohen S Yerushalmy-Feler A Chapnik N Tsameret S Anafy A . Circadian Clock gene disruption in white blood cells of patients with celiac disease. Biochimie. (2024) 219:51–4. doi: 10.1016/j.biochi.2023.07.020, PMID: 37524198

[98] Pagel R Bär F Schröder T Sünderhauf A Künstner A Ibrahim SM . Circadian rhythm disruption impairs tissue homeostasis and exacerbates chronic inflammation in the intestine. FASEB J. (2017) 31:4707–19. doi: 10.1096/fj.201700141RR, PMID: 28710114 PMC6159707

[99] Guo S-N Jiang X-Q Chen N Song S-M Fang Y Xie Q-M . Melatonin regulates circadian Clock proteins expression in allergic airway inflammation. Heliyon. (2024) 10:e27471. doi: 10.1016/j.heliyon.2024.e27471, PMID: 38496876 PMC10944242

[100] Sio YY Anantharaman R Lee SQ Matta SA Ng YT Chew FT . The Asthma-associated Per1-like domain-containing protein 1 (PerLD1) Haplotype Influences Soluble Glycosylphosphatidylinositol Anchor Protein (sGPI-AP) Levels in Serum and Immune Cell Proliferation. Sci Rep. (2020) 10:715. doi: 10.1038/s41598-020-57592-9, PMID: 31959860 PMC6970992

[101] Cao Q Gery S Dashti A Yin D Zhou Y Gu J . A role for the Clock gene, Per1 in prostate cancer. Cancer Res. (2009) 69:7619–25. doi: 10.1158/0008-5472.CAN-08-4199, PMID: 19752089 PMC2756309

[102] Gery S Komatsu N Kawamata N Miller CW Desmond J Virk RK . Epigenetic silencing of the candidate tumor suppressor gene Per1 in non-small cell lung cancer. Clin Cancer Research: Off J Am Assoc Cancer Res. (2007) 13:1399–404. doi: 10.1158/1078-0432.CCR-06-1730, PMID: 17332281

[103] Chen R Yang K Zhao NB Zhao D Chen D Zhao CR . Abnormal expression of Per1 circadian-Clock gene in oral squamous cell carcinoma. OncoTargets Ther. (2012) 5:403–7. doi: 10.2147/OTT.S38508, PMID: 23226027 PMC3513907

[104] Tian Y Xie Y Bai F Wang J Zhang D . Biological clock genes are crucial and promising biomarkers for the therapeutic targets and prognostic assessment in gastric cancer. J Gastrointestinal Cancer. (2024) 55:900–12. doi: 10.1007/s12029-024-01028-4, PMID: 38427147

[105] Liu YF Hao J Yuan GL Wei MY Bu YH Jin TT . Per1 as a tumor suppressor attenuated in the Malignant phenotypes of breast cancer cells. Int J Gen Med. (2021) 14:7077–87. doi: 10.2147/IJGM.S328184, PMID: 34712059 PMC8547972

[106] Chen M Zhang L Liu X Ma Z Lv L . Per1 is a prognostic biomarker and correlated with immune infiltrates in ovarian cancer. Front Genet. (2021) 12:697471. doi: 10.3389/fgene.2021.697471, PMID: 34220965 PMC8248530

[107] Gery S Komatsu N Baldjyan L Yu A Koo D Koeffler HP The circadian gene Per1 plays an important role in cell growth and DNA damage control in human cancer cells. Mol Cell. (2006) 22:375–82. doi: 10.1016/j.molcel.2006.03.038, PMID: 16678109

[108] Farshadi E van der Horst GTJ Chaves I . Molecular links between the circadian clock and the cell cycle. J Mol Biol. (2020) 432:3515–24. doi: 10.1016/j.jmb.2020.04.003, PMID: 32304699

[109] Sato F Nagata C Liu Y Suzuki T Kondo J Morohashi S . PerIOD1 is an anti-apoptotic factor in human pancreatic and hepatic cancer cells. J Biochem. (2009) 146:833–8. doi: 10.1093/jb/mvp126, PMID: 19675098

[110] Yang XM Wood PA Ansell CM Quiton DF Oh E-Y Du-Quiton J . The circadian Clock gene Per1 suppresses cancer cell proliferation and tumor growth at specific times of day. Chronobiology Int. (2009) 26:1323–39. doi: 10.3109/07420520903431301, PMID: 19916834

[111] Zhu L Wang QL Hu Y Wang F . The circadian gene per1 plays an important role in radiation-induced apoptosis and DNA damage in glioma. Asian Pacific J Cancer Prevention : APJCP. (2019) 20:2195–201. doi: 10.31557/APJCP.2019.20.7.2195, PMID: 31350984 PMC6745214

[112] Yao J He CQ Zhao WC Hu N Long DX . Circadian Clock and cell cycle: Cancer and chronotherapy. Acta Histochemica. (2021) 123:151816. doi: 10.1016/j.acthis.2021.151816, PMID: 34800857

[113] Bellet MM Stincardini C Costantini C Gargaro M Pieroni S Castelli M . The circadian protein per1 modulates the cellular response to anticancer treatments. Int J Mol Sci. (2021) 22:2974. doi: 10.3390/ijms22062974, PMID: 33804124 PMC8001324

[114] Han Y Meng F Venter J Wu N Wan Y Standeford H . miR-34a-dependent overexpression of Per1 decreases cholangiocarcinoma growth. J Hepatol. (2016) 64:1295–304. doi: 10.1016/j.jhep.2016.02.024, PMID: 26923637 PMC4874896

[115] Zhao B Nepovimova E Wu Q . The role of circadian rhythm regulator Pers in oxidative stress, immunity, and cancer development. Cell Communication Signaling : CCS. (2025) 23:30. doi: 10.1186/s12964-025-02040-2, PMID: 39825442 PMC11740368

[116] Li H-X Fu X-J Yang K Chen D Tang H Zhao Q . The Clock gene Per1 suppresses expression of tumor-related genes in human oral squamous cell carcinoma. Oncotarget. (2016) 7:20574–83. doi: 10.18632/oncotarget.7827, PMID: 26943040 PMC4991476

[117] Luo P Wu WL Jin F Long JH Li YY Zeng Y . Effects of biological clock gene Per1 on the invasion and migration of nasopharyngeal carcinoma cells CNE2 and the expression of JAK2-STAT3 pathway-related proteins. Modern Oncol. (2023) 31:2823–8. doi: 10.3969/j.issn.1672-4992.2023.15.011

[118] Kuo S-J Chen S-T Yeh K-T Hou M-F Chang Y-S Hsu NC . Disturbance of circadian gene expression in breast cancer. Virchows Archiv: Int J Pathol. (2009) 454:467–74. doi: 10.1007/s00428-009-0761-7, PMID: 19296127

[119] Gong X Tang H Yang K . Per1 suppresses glycolysis and cell proliferation in oral squamous cell carcinoma via the Per1/RACK1/PI3K signaling complex. Cell Death Dis. (2021) 12. doi: 10.1038/s41419-021-03563-5, PMID: 33723221 PMC7960720

[120] Wang J Huang Q Hu X Zhang S Jiang Y Yao G . Disrupting circadian rhythm via the per1–HK2 axis reverses trastuzumab resistance in gastric cancer. Cancer Res. (2022) 82:1503–17. doi: 10.1158/0008-5472.CAN-21-1820, PMID: 35255118 PMC9662874

[121] Wang ZX Wang H Wang ZJ He SM Jiang ZP Yan CP . Associated analysis of Per1/TUBB2B with endometrial cancer development caused by circadian rhythm disorders. Med Oncol. (2020) 37:90. doi: 10.1007/s12032-020-01415-4, PMID: 32926243

